# Lithium-sensing riboswitch classes regulate expression of bacterial cation transporter genes

**DOI:** 10.1038/s41598-022-20695-6

**Published:** 2022-11-09

**Authors:** Neil White, Harini Sadeeshkumar, Anna Sun, Narasimhan Sudarsan, Ronald R. Breaker

**Affiliations:** 1 Department of Molecular, Cellular and Developmental Biology, Yale University, New Haven, CT 06520-8103 USA; 2 Howard Hughes Medical Institute, Yale University, New Haven, CT 06520-8103 USA; 3 Department of Molecular Biophysics and Biochemistry, Yale University, New Haven, CT 06520-8103 USA

**Keywords:** Biochemistry, Genetics, Microbiology, Molecular biology

## Abstract

Lithium is rare in Earth’s crust compared to the biologically relevant alkali metal cations sodium and potassium but can accumulate to toxic levels in some environments. We report the experimental validation of two distinct bacterial riboswitch classes that selectively activate gene expression in response to elevated Li^+^ concentrations. These RNAs commonly regulate the expression of *nhaA* genes coding for ion transporters that weakly discriminate between Na^+^ and Li^+^. Our findings demonstrated that the primary function of Li^+^ riboswitches and associated NhaA transporters is to prevent Li^+^ toxicity, particularly when bacteria are living at high pH. Additional riboswitch-associated genes revealed how some cells defend against the deleterious effects of Li^+^ in the biosphere, which might become more problematic as its industrial applications increase.

## Introduction

Although most organisms are exposed to only modest amounts of lithium ions from the environment, elevated concentrations of this metal cation are toxic to certain eubacteria and eukaryotic species^1^. Seawater commonly maintains a Li^+^ concentration of ~ 25 μM^2^ but higher concentrations can occur near thermal vents. Concentrations in soil average ~ 20 mg kg^−1^, but amounts can vary by several orders of magnitude depending on regional geological characteristics^3,4^. Unfortunately, the molecular mechanisms of Li^+^ toxicity in general, and the reasons for its therapeutic effects on humans^5^, are poorly understood^6,7^.

Some evidence indicates that Li^+^ inhibits certain bacterial and eukaryotic phosphatase enzymes, thereby causing accumulation of the nucleotide 3́′-phosphoadenosine 5́′-phosphate (pAp)^8–11^ and the disruption of sulfur metabolism^12,13^. In addition, Li^+^ is known to affect signaling processes by disrupting the function of enzymes involved in phosphatidylinositol and glycogen metabolic pathways^6^. Li^+^ is also predicted to broadly affect enzymes that exploit Mg^2+^-ATP complexes^7^. Presumably, other molecular targets of Li^+^ exist wherein cation binding disturbs normal cellular functions.

One strategy to investigate the mechanisms of Li^+^ toxicity resistance is to discover how cells sense elevated concentrations. Previous discoveries of riboswitches that sense fluoride^14–16^ or the divalent heavy metal cations nickel and cobalt^17^ revealed genes whose protein products are expressed to help mitigate the deleterious effects of these ions. Similarly, biological systems are likely to have evolved mechanisms to overcome the adverse effects of Li^+^, and these might be revealed by identifying links between Li^+^ sensing and toxicity mitigation factors.

Herein we report the experimental validation of two distinct classes of Li^+^-sensing bacterial riboswitches that regulate genes whose protein products presumably mitigate Li^+^ toxicity. Specifically, we used bioinformatic, genetic, and biochemical analyses to provide support for the function of these RNAs as direct sensors of Li^+^ that activate gene expression only when this ion is high in concentration. Our findings indicate that the primary strategy used by bacteria to overcome the toxic effects of Li^+^ is to eject the ion from cells.

## Results and discussion

### Structured RNA motifs discovered by comparative sequence analysis were riboswitch candidates for elemental ions

**Figure 1:**
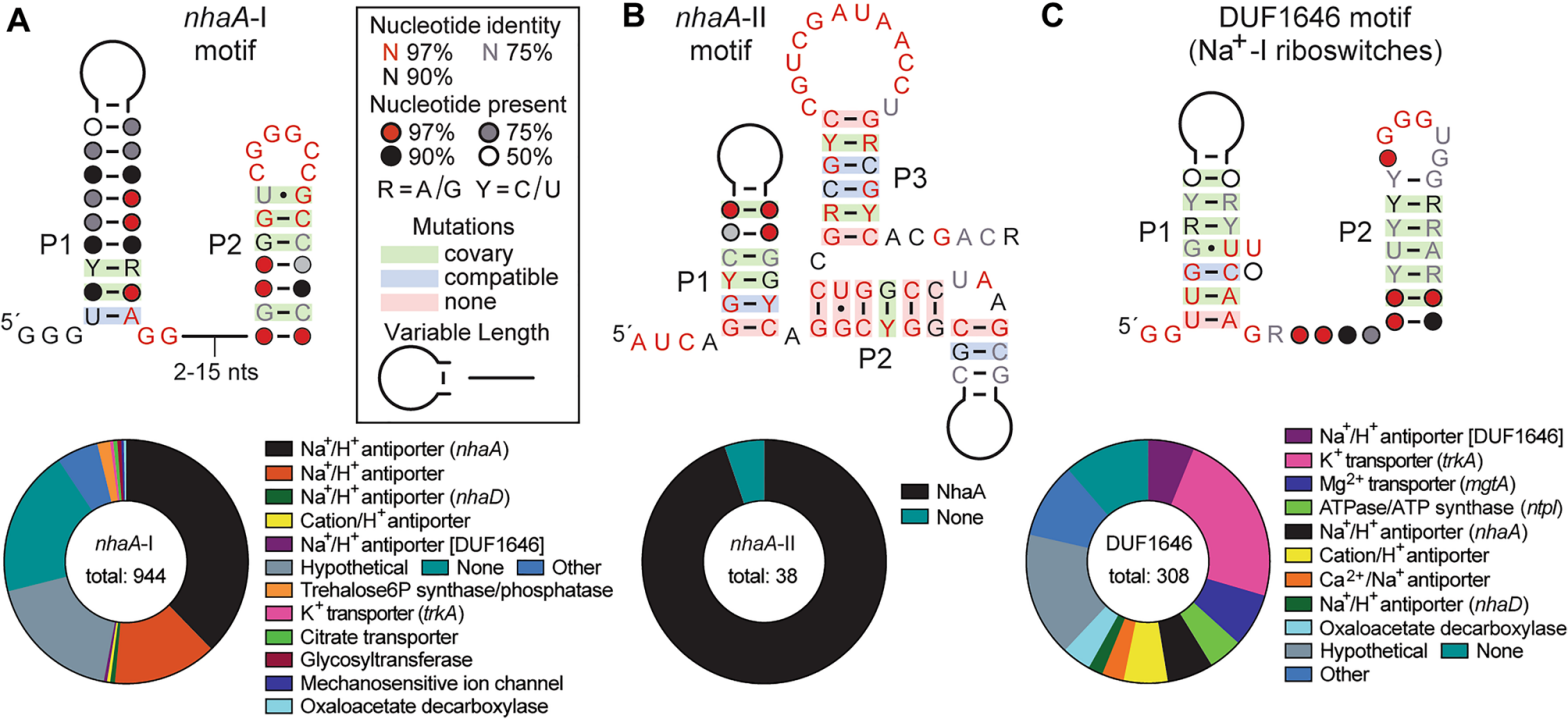
Three distinct classes of riboswitch candidates for alkali metal cations. (Top) Sequence and secondary structure models for the *nhaA*-I, *nha*A-II and DUF1646 motif RNAs. Consensus models for *nhaA*-I and *nha*A-II were generated based on updated sequence alignments (see supplemental files). The consensus model for DUF1646 (Na^+^-I riboswitch aptamer) was prepared as described elsewhere^19^. (Bottom) Annotated functions of proteins encoded by genes (parentheses) commonly associated with the three riboswitch candidates (see also Supplementary Table I in the Supplement). Data are based on the total number of non-redundant representatives as noted.

Three structured, noncoding RNA (ncRNA) classes were previously discovered that all commonly associate with genes whose protein products are predicted to be monovalent ion transporters^18^. These ncRNA classes, called the *nhaA*-I, *nhaA*-II, and DUF1646 motifs (Fig. 1A–C, top), were considered riboswitch candidates, wherein the conserved domain of each RNA functions as an aptamer that binds a ligand to regulate expression of the adjacent gene. Given the gene associations common for the three motifs (Fig. 1A–C, bottom), we speculated that they would sense one or more biologically relevant alkali ions to trigger riboswitch-mediated gene control. Indeed, we have established that DUF1646 motif RNAs function as highly selective riboswitches for Na^+^ ions^19^. Representative DUF1646 motif RNAs (hereafter called Na^+^-I riboswitches) were shown to activate gene expression in response to elevated Na^+^ concentrations, thereby regulating the expression of various genes either known or predicted to be relevant to sodium transport or utilization (Fig. 1C). These RNAs bind Na^+^ with 1-to-1 stoichiometry and strongly reject all other mono- and divalent alkali and alkaline earth cations tested^19^.

The consensus sequence and secondary structure model for Na^+^-I riboswitch aptamers (Fig. 1C) is similar to that for *nhaA*-I motif RNAs (Fig. 1A). Despite their similarities, the consensus models exhibit distinct conserved features in the lower portion of the first base-paired region (P1) and in the loop of P2 that served as the basis for sorting representatives into two groups. Some genes associated with Na^+^-I riboswitches, such as DUF1646, *nhaD*, and *oadG*, are also occasionally associated with *nhaA*-I motif RNAs. Thus, we speculated that *nhaA*-I motif RNAs might sense a ligand that is chemically similar to Na^+^, such as a different alkali metal cation. Moreover, although the *nhaA*-I motif shares no similarity in either sequence or architecture to the *nhaA*-II motif (Fig. 1B), they both most frequently associate with *nhaA* genes. This observation strongly suggested that *nhaA*-I and *nhaA*-II motifs form distinct RNA structures that sense the same ligand.

### Members of the ***nhaA***-I and ***nhaA***-II motif classes function as Li^+^-specific genetic switches

NhaA proteins (pfam06965, pfam07399 and COG3004) are known to function as Na^+^/H^+^ antiporters, and these membrane-localized proteins are generally implicated in Na^+^ homeostasis^20,21^, along with another demonstrated Na^+^/H^+^ protein class called NhaB^22^. However, previous biochemical assays^21,23,24^ reveal that NhaA proteins robustly transport both Na^+^ and Li^+^. In *Escherichia coli*, it was demonstrated that deleting the *nhaA* gene results in increased Li^+^ toxicity, whereas deleting the *nhaB* gene alone has no effect on Li^+^ sensitivity^25^. Furthermore, expression of the bacteria NhaA protein confers tolerance for Li^+^, but not Na^+^, in *Saccharomyces cerevisiae*^26^. Because the *nhaA*-I and *nhaA*-II motif RNAs are associated with *nhaA* genes and never *nhaB* genes, we considered the possibility that these RNAs might function as selective Li^+^-sensing riboswitches.

**Figure 2:**
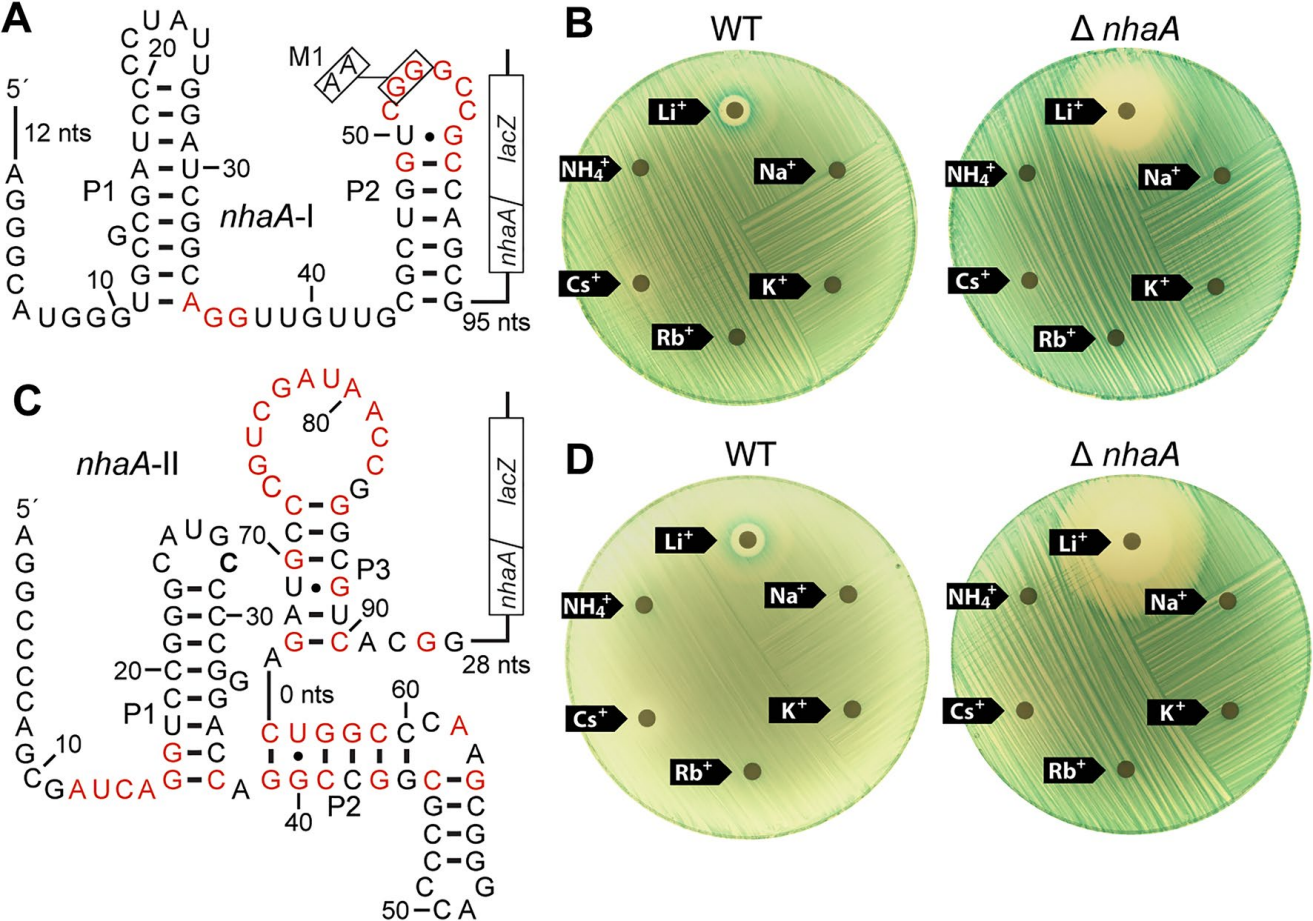
Li^+^ triggers gene expression mediated by bacterial *nhaA*-I and *nhaA*-II motif RNAs. (**A**) A *nhaA*-I riboswitch-reporter fusion construct was created by fusing a representative associated with the *nhaA* gene of *Azorhizobium caulinodans* to a β-galactosidase gene (*lacZ*). Red nucleotides correspond to the highly conserved nucleotides characteristic of this RNA motif class (Fig. 1A). Boxed nucleotides identify locations of G-to-A mutations present in construct M1, which is a mutant riboswitch RNA used in several subsequent experiments. (**B**) Agar-diffusion assays were conducted with *E. coli* cells (either wild type [WT] or a *nhaA* gene deletion [Δ*nhaA*] strain) carrying the *nhaA*-I reporter construct in A. Cells were spread on LBK (pH 9.1) agar media containing X-gal, and filter disks using 10 μL applications of 5 M chloride salts of various monovalent ions as indicated, except that reduced concentrations of KCl (3 M) and RbCl (0.5 M) were used due to limited solubility. (**C**) A *nhaA*-II riboswitch-reporter fusion construct was created by fusing a representative associated with the *nhaA* gene of *Brevundimonas subvibrioides* to *lacZ*. Additional annotations are as described for A. (**D**) Agar-diffusion assays with various cations were conducted with the *nhaA*-II reporter as described in C. Additional annotations and details are as described for B.

To assess the ligand binding and gene regulation functions of *nhaA*-I motif RNAs, we first prepared a genetic fusion (translational) between a β-galactosidase (*lacZ*) reporter gene and the riboswitch candidate based on the *nhaA*-I motif representative from *Azorhizobium caulinodans* (Fig. 2A). Transcription is driven by a heterologous (*Bacillus subtilis lysC*) promoter known to be constitutively active^27,28^. This construct, evaluated in surrogate *E. coli* cells grown on LBK agar plates at pH 9.1 using agar diffusion assays (see Materials and Methods), yielded low reporter gene expression in the absence of added Li^+^ and robust gene expression when cells were experiencing Li^+^ toxicity (Fig. 2B).

Furthermore, mutant *E. coli* cells lacking the gene coding for the native NhaA protein (Δ*nhaA*) were sensitive to lower concentrations of Li^+^ and exhibited higher reporter gene expression even when Li^+^ was not supplemented in the growth medium. This result suggested that the Δ*nhaA* strain could not efficiently expel Li^+^ that accumulated in cells even when only trace amounts were present in the medium. These gene regulation and toxicity characteristics were specific for Li^+^ because no other monovalent ion tested triggered riboswitch function or inhibited cell growth (Fig. 2B).

Likewise, a reporter-fusion construct integrating a representative of the *nhaA*-II riboswitch candidate (Fig. 2C) yielded similar reporter expression results (Fig. 2D). These findings were consistent with the hypothesis that at least two distinct Li^+^-responsive riboswitch classes exist among species of bacteria, wherein the RNAs can sense toxic levels of this alkali metal cation and activate the expression of NhaA proteins. Thus, Li^+^-responsive riboswitches and NhaA proteins appeared to be key components of Li^+^ toxicity defense responses in some bacterial species. Hereafter, we refer to these riboswitch classes as Li^+^-I (*nhaA*-I motif) and Li^+^-II (*nhaA*-II).

Notably, reporter assays for both *nhaA*-I and *nhaA*-II constructs yielded patterns of faint blue color nearest to the filter disk wherein Li^+^ was applied, and a more intense blue ring farther from the disk. This pattern could be due to a reduction in cell growth caused by Li^+^ toxicity (faint blue) near the disk, and Li^+^-triggered induction of gene expression mediated by the riboswitch at a distance where cells are less stressed. Similar patterns are observed for other validated riboswitches when evaluated using agar diffusion assays^29^.

**Figure 3:**
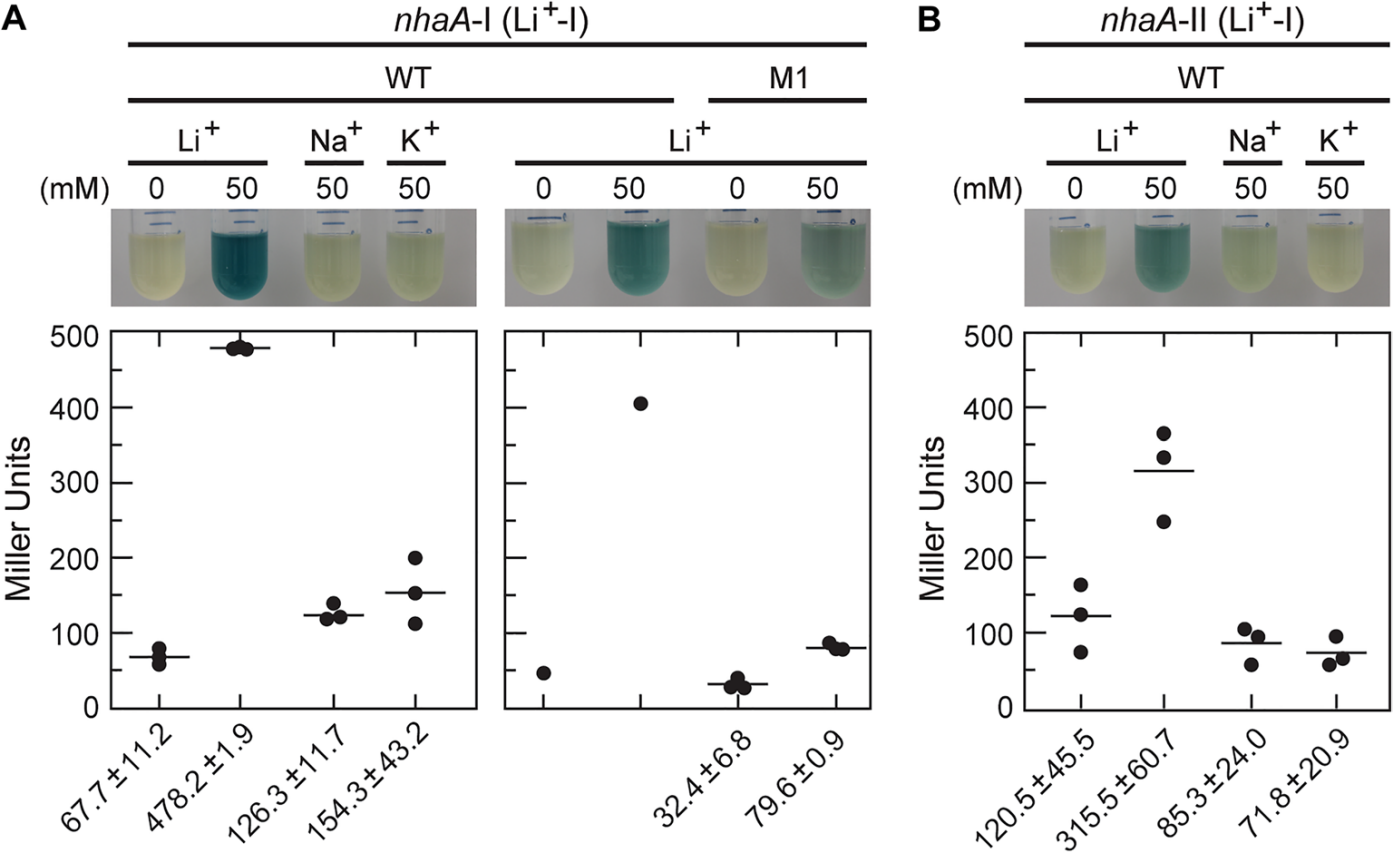
Quantitation of gene expression mediated by representative Li^+^-I and Li^+^-II riboswitches. (**A**) Left: *E. coli* cells carrying the WT *nhaA*-I (Li^+^-I) riboswitch-reporter fusion construct (see Fig. 2A**)** grown in low salt LB medium at pH 9 either without or with supplementation with 50 mM LiCl, NaCl, or KCl as indicated. Either X-gal (top) or ONPG (bottom), respectively, was added after overnight incubation of cultures to visualize or quantify β-galactosidase reporter activity. Right: *E. coli* cells carrying the WT *nhaA*-I (Li^+^-I) or M1 *nhaA*-I (Li^+^-I) riboswitch-reporter fusion construct (see Fig. 2A**)** cultured in low salt LB medium at pH 9 either without or with supplementation of 50 mM LiCl. The mean and standard deviation values are presented for experiments conducted in triplicate (n = 3). (**B**) *E. coli* cells carrying the WT *nhaA*-II (Li^+^-II) riboswitch-reporter fusion construct (Fig. 2C) were cultured in low salt LB medium at pH 9.0 without or with supplementation of 50 mM LiCl, NaCl, or KCl as indicated.

Quantitative reporter gene assays were also conducted with the Li^+^-I and Li^+^-II riboswitch-reporter fusion constructs described above. *E. coli* cells carrying the WT *nhaA*-I (Li^+^-I) riboswitch-reporter fusion construct (Fig. 2A) and cultured in rich medium at pH 9.0 exhibited robust reporter gene expression when exposed to 50 mM Li^+^, but these cells were largely unresponsive to media supplementation with 50 mM Na^+^ or K^+^ (Fig. 3A, left). Furthermore, the introduction of mutations that altered two nucleotides of the highly conserved loop region (Fig. 2A, construct M1: G52A, G53A) substantially diminished reporter gene expression in response to Li^+^ addition (Fig. 3A, right). This finding was consistent with the observation that the M1 construct exhibited poorer affinity for Li^+^ compared to the WT construct (see below). *E. coli* cells carrying the WT *nhaA*-II (Li^+^-II) riboswitch-reporter fusion construct (Fig. 2B) exhibited a similar selective response to Li^+^ (Fig. 3B). These findings indicated that Li^+^-I and Li^+^-II riboswitches most likely use their highly conserved sequence and structural elements to respond exclusively to Li^+^, which is also consistent with the fact that cells become more sensitive only to Li^+^ when a gene most associated with Li^+^ riboswitches was deleted (Δ*nhaA* strain) (Fig. 2).

### Biochemical assays confirm ligand responsiveness by Li^+^-I and Li^+^-II riboswitches

**Figure 4:**
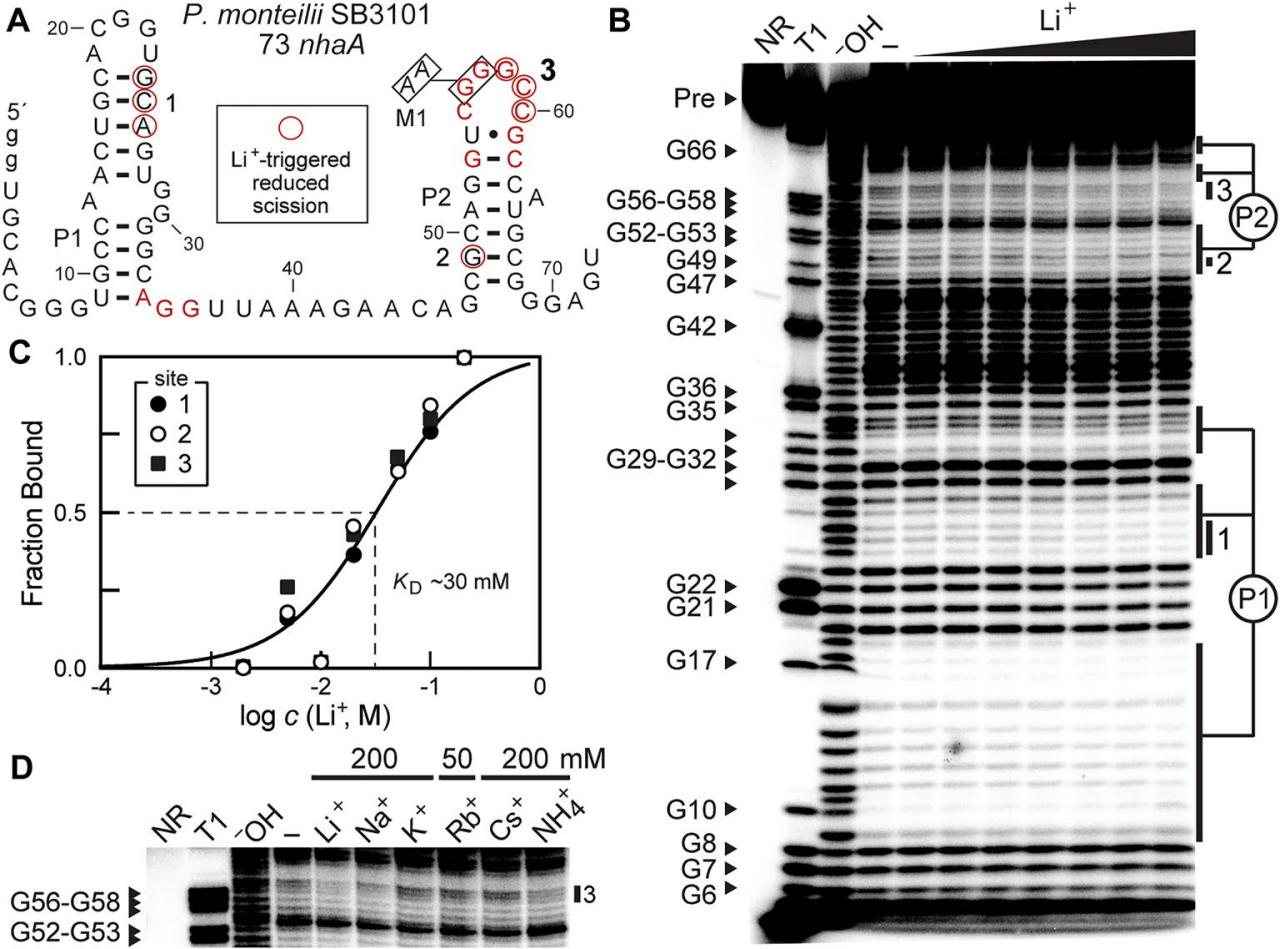
Li^+^ induces structural modulation of a Li^+^-I aptamer. (**A**) Sequence and secondary structure model for the Li^+^-I riboswitch aptamer construct 73 *nhaA* derived from the *nhaA* gene from *P. monteilii*. Lowercase g letters identify non-native nucleotides added to facilitate production by in vitro transcription and red nucleotides are highly conserved in *nhaA*-I motif RNAs as depicted in Fig. 1A. Boxed nucleotides at positions 56 and 57 were mutated to A nucleotides in construct M1. Nucleotides circled in red are among those that undergo reduced spontaneous cleavage during in-line probing assays upon the addition of Li^+^, as determined from the autoradiogram depicted in B. (**B**) PAGE analysis of in-line probing reactions with 5′ ^32^P-labeled 73 *nhaA* RNA in the absence of Li^+^ (–), or in the presence of Li^+^ concentrations ranging from 2 to 200 mM. NR, T1 and ^–^OH identify RNAs subjected to no reaction, partial digestion with RNase T1 (cleaves after G nucleotides) and partial digestion with hydroxide (cleaves after each nucleotide). Bands corresponding to RNAs carrying a 3′ G nucleotide are identified according to the numbering system in A. A lower contrast version of the gel image is presented in Supplementary Fig. I in the Supplement. (**C**) Plot of the estimated fraction of RNAs bound to Li^+^ versus the logarithm of the Li^+^ concentration. Fraction bound values were estimated by quantifying band intensities at sites 1, 2 and 3 in B, which correspond to the nucleotides annotated with red circles in A. Note that fraction bound values were set to 1 at the maximum Li^+^ concentration tested because the band intensities are near zero (maximal possible suppression). The solid line depicts a theoretical 1-to-1 binding curve with a *K*_D_ of 30 mM. (**D**) PAGE analysis of in-line probing assays as described for B wherein reactions were supplemented with 200 mM of the ions indicated, except Rb^+^ was tested at 50 mM. Reduced band intensities at site 3 depicted here are indicative of ion binding. A lower contrast version of the full gel image and comparisons of sites 1, 2 and 3 are presented in Supplementary Fig. IV in the Supplement.

Selective cation recognition by Li^+^-I riboswitches was also established using in-line probing^30,31^, which is a method that exploits the chemical instability of RNA to monitor folding changes in response to ligand binding. For example, an RNA construct called 73 *nhaA* (Fig. 4A), whose design is based on a representative Li^+^-I aptamer associated with the *nhaA* gene of the bacterium *Pseudomonas monteilii*, produced a spontaneous degradation pattern (Fig. 4B) that was largely consistent with the proposed consensus secondary structure model^18^. Even though 2 mM Mg^2+^ and 100 mM K^+^ were present in the in-line probing reaction mixtures, the RNA underwent changes in band intensity in several locations upon the addition of Li^+^, most noticeably in the linker region between stems P1 and P2 (Supplementary Fig. I in the Supplement).

Three sites of band intensity changes, chosen because these RNA fragments are produced upon cleavage within sequence or structural regions typical of this aptamer class, were evaluated to assess the ligand-binding characteristics of the RNA. Albeit modest, band intensity changes at these sites occurred in a concerted fashion, yielding a ligand binding curve consistent with a 1-to-1 interaction and an apparent dissociation constant (*K*_D_) of ~ 30 mM (Fig. 4C). Similar results were observed for a Li^+^-I riboswitch aptamer from the bacterium *Ralstonia eutropha* (Supplementary Fig. II in the Supplement), which exhibited a *K*_D_ of no lower than ~ 20 mM for Li^+^.

In contrast, Na^+^ began to induce changes in band intensities for the 73 *nhaA* RNA construct only at concentrations ~ tenfold higher (Supplementary Fig. III in the Supplement), indicating that Li^+^ is the preferred ligand. In-line probing reactions were also used to survey the effects of several other alkali metal ions and ammonium (NH_4_^+^) at 200 mM or Rb^+^ at 50 mM (Fig. 4D, Supplementary Fig. IV in the Supplement). The RNA aptamer strongly rejected K^+^, Rb^+^ and Cs^+^, and exhibited only poor affinity for Na^+^ and NH_4_^+^. Relatively low cellular concentrations of ammonium^32^ and Na^+^ under non-osmotic stress conditions^33^ in combination with their poorer riboswitch affinities likely prevent these ions from triggering gene regulation through Li^+^ riboswitches. Furthermore, mutation of strictly conserved G nucleotides to A nucleotides (construct M1: G56A, G57A) (Fig. 4A) greatly weakened the response of the riboswitch reporter construct to Li^+^ (Supplementary Fig. V in the Supplement), indicating that these positions are important for ligand binding. This latter observation is consistent with the fact that the M1 construct exhibited substantially reduced responsiveness to Li^+^ in riboswitch-reporter fusion assays (Fig. 3B, right).

### Rare variants of Li^+^-I riboswitches exhibit altered ion specificity and are robustly triggered by Na^+^

**Figure 5:**
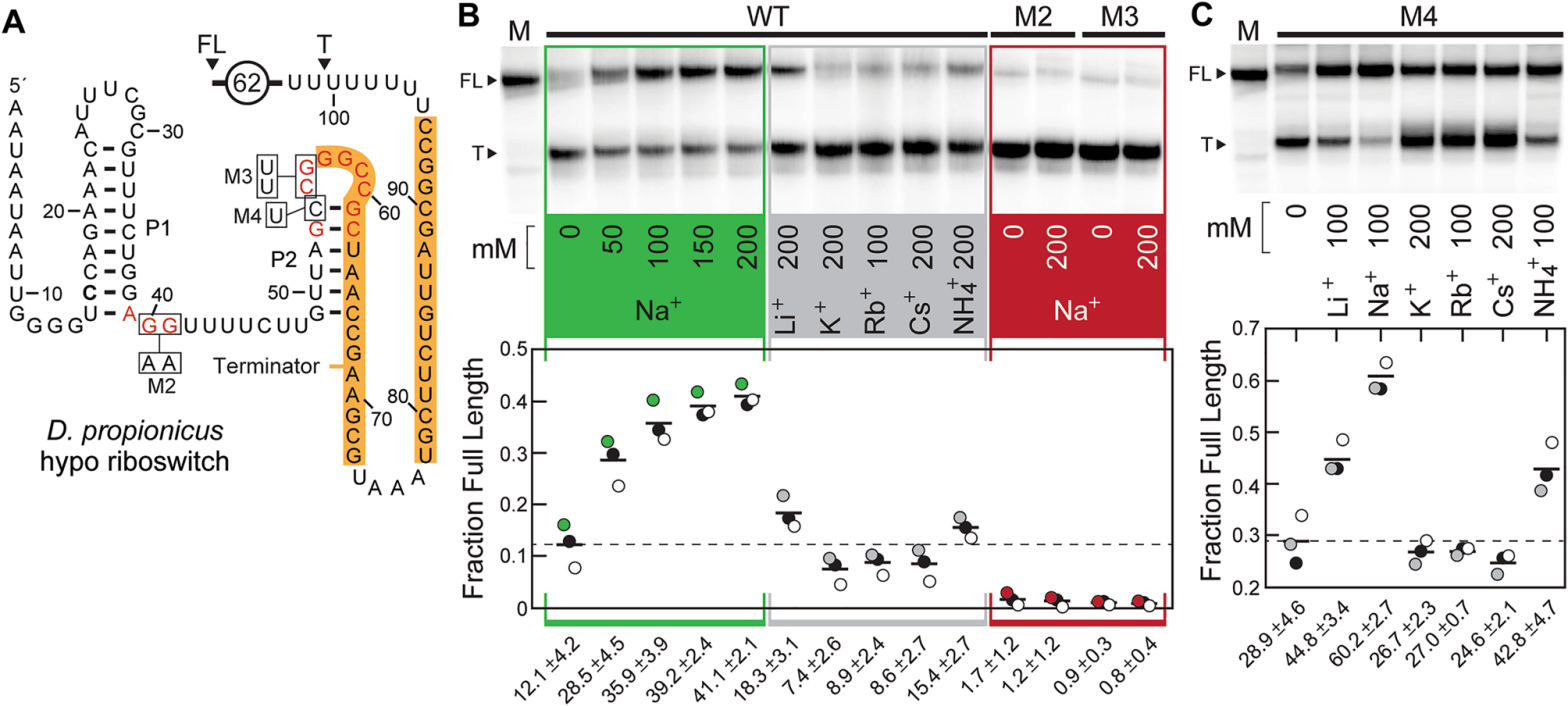
Variants of Li^+^-I riboswitches naturally respond to Na^+^. (**A**) Sequence and secondary structure model for a Na^+^-sensing riboswitch associated with a gene annotated as “hypothetical” (Supplementary Table I in the Supplement) from *D. propionicus*. Orange shading indicates alternative pairing that is predicted to form an intrinsic terminator stem. Predicted terminated (T) and full-length (FL) RNA transcripts are denoted with arrowheads. Other annotations are as described for Figs. 2 and 3. (**B**) Top: PAGE autoradiogram of an example single-round transcription termination assay series with a DNA template producing the *D. propionicus* construct depicted in A. Bands corresponding to FL and T transcripts identified in A are denoted. Transcription reactions were supplemented with the ions as indicated, and M identifies the lane loaded with a marker approximating FL RNA. Bottom: plot of the fraction of FL transcripts for each transcription reaction conducted in triplicate, where the circle colors matching the panels were derived from the gel shown. The dashed line represents the average fraction FL in the absence of additional ion supplementation above the 4 mM Na^+^ initially present in the transcription reaction. An uncropped version of the gel image is presented as Supplementary Fig. VIII in the Supplement. (**C**) Top: PAGE autoradiogram of an example transcription termination assay series using RNA construct M4, which carries a C54U mutation that represents the nucleotide at this position most commonly observed with *nhaA*-I motif RNAs. Bottom: Plot of the fraction of FL transcripts for each transcription reaction conducted in triplicate (n = 3), where the numbers indicate the mean and standard deviation. Additional annotations are as described for B. An uncropped version of the gel image is presented as Supplementary Fig. IX in the Supplement.

Although we speculate that most Li^+^-I and Li^+^-II riboswitches regulate gene expression by controlling ribosome access to the ribosome binding site (RBS) of the adjoining open reading frame, rare variants of Li^+^-I riboswitch aptamers carry an intrinsic terminator stem^34,35^ as an expression platform^36^. Thus, we speculated that these unusual variants might bind the ligand and prevent transcription termination from occurring upstream of the protein coding region of the messenger RNA as a mechanism to regulate gene expression. Indeed, we observed that a representative of this variant collection associated with a hypothetical gene from the bacterium *Desulfobulbus propionicus* (Fig. 5A) robustly activated transcription upon ligand binding as determined by in vitro transcription assays, whereas mutants M2 and M3 failed to respond (Fig. 5B). However, surprisingly, the construct chosen for this analysis was strongly activated upon the addition of Na^+^ and was only weakly triggered by Li^+^.

To further investigate this unexpected outcome, we hypothesized that some RNAs originally included in the *nhaA*-I motif collection carry nucleotide changes that switch the specificity from Li^+^ to Na^+^. Natural riboswitch specificity changes are well demonstrated to occur through evolution^37–39^. Consistent with this hypothesis, a construct carrying 71 nucleotides encompassing the variant *D. propionicus* (called 71 hypo, Supplementary Fig. VIA in the Supplement) exhibited structural modulation only with Na^+^ among the alkali metal cations tested (Supplementary Fig. VIB in the Supplement). In addition, the estimated *K*_D_ value for Na^+^ binding was in the low mM range (Supplementary Fig. VIC,D in the Supplement), which is similar to the affinities measured for Na^+^-I riboswitches described previously^19^.

The hypothesis of a ligand specificity change for some *nhaA*-I motif RNAs was further explored by examining the aptamers grouped into this collection that also associate with intrinsic terminator expression platforms. These variant riboswitches carrying terminator stems were found only in species of Deltaproteobacteria, where they are located upstream of various genes coding for proteins of unknown function. These gene associations were not found with the remaining RNAs that conform to either Li^+^-I or Li^+^-II riboswitch classes, suggesting that the variant RNAs sense a ligand other than Li^+^.

Furthermore, most *nhaA*-I motif RNAs carry a U nucleotide corresponding to nucleotide position 54 of the RNA constructs derived from *D. propionicus* (Fig. 5A, Supplementary Fig. VIA in the Supplement), whereas the natural variant riboswitch carries a C nucleotide at this position. By making a single C54U mutation in the original riboswitch sequence (M4, Fig. 5A), the ion responsiveness during transcription was broadened to include Li^+^, Na^+^, and NH_4_^+^ (Fig. 5C). These results indicate that some RNAs originally grouped with the *nhaA*-I motif RNAs carry nucleotide changes that alter their ligand specificity from Li^+^ to Na^+^. Given that these Na^+^-sensing variant aptamers described herein carry substantial differences in the consensus model compared to Na^+^-I riboswitches described previously^19^, we have named these rare variants Na^+^-II riboswitches.

## Concluding remarks

The discovery of riboswitches for Li^+^ reveals the only known mechanisms by which bacterial cells sense and mitigate high concentrations of this toxic alkali metal cation. The demonstrated selectivity of both Li^+^ riboswitch classes in vivo (Figs. 2, 3) highlights the question of how RNAs form binding pockets that favor Li^+^ binding over other monovalent ions. The repetitive negatively charged phosphodiester backbone of RNA clearly creates a favorable ionic environment for the association of cations. Furthermore, alkali metal cations are well known to stabilize various structural features of folded RNAs^40^. These characteristics seem to make RNA well suited to form structures that selectively bind monovalent ions. Indeed, engineered RNA aptamers have been identified that bind vitamin B_12_ only in the presence of Li^+^, which provides a precedence for the selective binding of this cation^41^.

We considered the possibility that bacterial species living in high Li^+^ concentration environments might be more likely to carry these riboswitches. The *nhaA*-I RNA motif is present in diverse species of Alpha-, Beta-, Gamma-, and Deltaproteobacteria, whereas the *nhaA*-II RNA motif appears to be present only in some species of Gammaproteobacteria. At present, we have not found a notable bias in favor of these riboswitches in organisms that are known to live in environments high in lithium. Thus, it seems likely that bacteria living in many ordinary environments must have mechanisms to sense and respond to lithium toxicity, suggesting that additional sensors for Li^+^ await discovery.

Most genes associated with Li^+^ riboswitches appear to be cation transporters (Fig. 1, Supplementary Table I in the Supplement), and our findings strongly indicated that the primary function of some NhaA proteins is to export Li^+^ as concentrations approach toxic levels. It is notable that cells become more sensitive to high Li^+^ concentrations under alkaline conditions (Supplementary Fig. VII in the Supplement), and we speculate that this is a result of the use of Na^+^ gradients to power solute transport. At high pH, cells can harness the energy present in ion gradients to import various solutes^42^. Excess cellular Na^+^ can then be used by Na^+^-H^+^ antiporters to adjust internal pH. If this import system is imperfect, then Li^+^ might also enter cells predominantly at high pH because it mimics the function of Na^+^. Under these circumstances, Li^+^ riboswitches and NhaA proteins could selectively sense and preferentially expel excess Li^+^. Given that NhaA proteins are also ion-proton antiporters, this action would both alleviate Li^+^ toxicity and contribute to reducing cellular pH.

Intriguingly, among bacterial sequences generated from an environmental bacterial sample, we identified several instances wherein riboswitches conforming to the Li^+^-I class reside in tandem. Such tandem arrangements of riboswitches for the same ligand have been observed previously, and they usually function to reduce the ligand concentration range needed to maximally trigger changes in gene expression^43^. However, tandem riboswitch arrangements wherein the aptamers sense different ligands yield genetic versions of Boolean logic gates^44,45^, wherein gene expression depends on the concentrations of two different chemical inputs. The genes associated with these tandem Li^+^-I riboswitches are generally related to cation transport and osmotic stress, and it was not determined if the aptamers are selective for Li^+^ or Na^+^. In addition, one example of a triple riboswitch arrangement was observed preceding a *nhaD* gene, wherein each aptamer is followed by an intrinsic terminator stem. Again, the ligand specificity of each riboswitch is unknown, but this appears likely to activate the expression of an NhaD cation/H^+^ antiporter protein in response to very small changes in ligand concentration.

It is known that fluoride riboswitches commonly upregulate the expression of genes for enzymes that are inhibited by this toxic anion. This action should help maintain the metabolic flux through pathways where key enzymes have reduced activity due to F^−^ inhibition. However, most genes coding for proteins previously implicated^7–13^ in the biological/toxic effects of Li^+^ are not listed among those associated with the two Li^+^-sensing riboswitch classes. Intriguingly, a gene coding for trehalose-6-phosphate synthase is occasionally associated with Li^+^-I riboswitches^19^ (Supplementary Table I in the Supplement). It has been established^46,47^ that trehalose biosynthesis in some eukaryotes is altered by exposure to Li^+^, and thus some bacteria might use Li^+^ riboswitches to regulate trehalose biosynthesis when concentrations of the cation are high. Alternatively, these riboswitches might be variants that sense Na^+^, and regulation might be part of an osmotic stress process involving trehalose.

Other genes associated with Li^+^ riboswitches might provide clues regarding the molecular targets relevant to Li^+^ toxicity. However, the rarity of gene associations beyond *nhaA* suggests that the deleterious effects of Li^+^ cannot be easily overcome by upregulating its molecular targets. This conclusion is consistent with the possible action of Li^+^ in eukaryotes, which has been proposed to induce its effects by binding to many targets^7^. If true, the most practical way for most bacteria to overcome Li^+^ toxicity might be to lower its concentration by ejection from the cell.

## Methods

### Chemicals and biochemical

Lithium chloride was purchased from Acros Organics and ammonium chloride was purchased from Macron Fine Chemicals. [α-^32^P]UTP and [γ-^32^P]ATP were purchased from PerkinElmer. All other chemicals and synthetic DNA oligonucleotides were purchased from Sigma-Aldrich. RNase T1 was purchased from Roche and all other enzymes were purchased from New England Biolabs. All salts were of 99% or greater purity.

### Bioinformatics analyses

Consensus models for the *nhaA*-I (Fig. 1A) and *nhaA*-II (Fig. 1B) motifs were updated by first conducting searches for additional representatives using CMFinder and Infernal 1.1 algorithms^48,49^ as described previously^18^. The consensus model for DUF1646 (Na^+^-I riboswitch aptamers) is depicted (Fig. 1C) as published previously^19^. Searches were conducted against the RefSeq 80 genomic sequence database and a collection of microbial environmental sequences (env12). Consensus sequence and secondary structure models were created with the computer program R2R^50^, and covariation annotations were defined using R-scape^51^.

Gene associations, defined as the first gene located immediately downstream of each RNA motif representative, were established by manual examination of each representative. In some instances, gene identities were established by using the NCBI Basic Local Alignment Search Tool (BLAST)^52^ to identify annotated protein homologs with known functional annotations. Pie charts were then generated to present the distributions of genes associated with each motif class.

### RNA constructs

Synthetic DNA oligonucleotides (Supplementary Table II in the Supplement) were used to prepare RNA transcription templates by overlap extension using SuperScript II reverse transcriptase following the manufacturer’s protocol (Thermo Fisher Scientific). The resulting double-stranded DNA templates were transcribed using T7 RNA polymerase in 50 μL reactions (80 mM HEPES [pH 7.5 at ~ 20 °C], 24 mM MgCl_2_, 2 mM spermidine, 40 mM DTT) incubated overnight at 37 °C. RNA products were separated using denaturing (8 M urea) polyacrylamide gel electrophoresis (PAGE), and the gel portions containing the desired RNAs were excised, crushed, and incubated in 350 μL crush-soak solution (200 mM NH_4_Cl, 10 mM Tris–HCl [pH 7.5 at ~ 20 °C], 1 mM EDTA) for 30 min at ~ 20 °C. RNAs were recovered from gel extracts by precipitation with cold ethanol followed by centrifugation. The resulting pellet was dried under vacuum and resuspended in deionized water (dH_2_O). RNA solutions were quantified by measuring the absorbance at 260 nm and calculating molarity using extinction coefficients estimated for each product.

RNAs (75 pmol) were dephosphorylated using rAPid Alkaline Phosphatase (Roche) following the manufacturer’s protocol. 10 pmol of each resulting RNA was 5′ ^32^P-labeled using T4 polynucleotide kinase in a 20 μL reaction containing 5 mM MgCl_2_, 25 mM CHES (pH 9.0), 3 mM DTT, and 20 μCi [γ-^32^P]-ATP. Radiolabeled RNAs were purified by PAGE, and salts were removed by performing three dH_2_O washes through an Amicon Ultra-0.5 centrifugal filter unit (3 KDa molecular weight cut-off).

### In-line probing assays

In-line probing assays were conducted as described previously^30,31^, except that the concentration of MgCl_2_ used was 2 mM. ^32^P-labeled RNAs were incubated with candidate ligands for 1 min at 75 °C before the addition of room-temperature in-line probing buffer (50 mM Tris–HCl [pH 8.3 at ~ 20 °C], 2 mM MgCl_2_, 100 mM KCl). The resulting mixtures were incubated at room temperature for between 40 and 70 h. Denaturing 10% PAGE was used to separate the resulting RNA degradation products, which were visualized using a Typhoon FLA 9500 Molecular Scanner (GE Healthcare). Product band intensities were quantified using ImageQuant software and bands whose intensities are modulated by ligand introduction were used for estimating *K*_D_ values for RNA-ligand interactions. The resulting intensity values were scaled to a fraction between 0 and 1 (greatest change), then plotted against the logarithm of the ligand concentration. Apparent *K*_D_ values were calculated using a sigmoidal-dose response equation in GraphPad Prism 8.

### Riboswitch reporter assays

Riboswitch reporter fusion constructs for both *nhaA*-I and *nhaA*-II motifs were prepared by PCR amplification of synthetic DNA constructs (Supplementary Table II in the Supplement) with the *lysC* promoter from *B. subtilis* directly preceding the riboswitch sequence of interest. The resulting DNAs were digested with the appropriate restriction enzyme and ligated into the pRS414 reporter vector (gift from R.W. Simons, UCLA). Plasmids were used to transform the WT *E. coli* BW25113 strain and its isogenic derivative BW25113 (*ΔnhaA::kan*). *E. coli* cells were obtained from the Coli Genetics Stock Center at Yale University.

For agar-diffusion assays, cells with the desired reporter plasmid were grown overnight in LBK media (standard LB media with sodium replaced with 100 mM KCl). LBK agar plates were buffered with AMPSO (100 mM, pH adjusted with KOH) and contained X-gal (5-bromo-4-chloro-3-indolyl β-d-galactopyranoside; 100 µg mL^−1^) and carbenicillin (100 µg mL^−1^). The choice of LBK and buffer was based on established methods^53^. Assays were conducted at pH 9.0 or 9.1 (100 mM K^+^, 100 mM AMPSO) as noted for each experiment. 10 μL of 5 M LiCl, 5 M NaCl, 3 M KCl, 0.5 M RbCl, 5 M CsCl, or 5 M NH_4_Cl was added to filter disks as indicated. The concentrations of stock salt solutions were chosen based on solubility. Plates were incubated overnight at 37 °C and were then photographed to record growth and β-galactosidase activity.

Liquid cultures inoculated with *E. coli* strains carrying riboswitch reporter fusion constructs were used to conduct visual (X-gal) or quantitative (ONPG) β-galactosidase assays. Cells with the indicated reporter plasmid were grown overnight in low sodium LB (yeast extract and tryptone). Then 100 μL of the overnight culture was subcultured in low sodium LB buffered at pH 9.0 with 100 mM AMPSO (pH adjusted with KOH) containing carbenicillin (100 µg mL^−1^) plus the 50 mM of the supplemented ion as indicated. Higher concentrations of Li^+^ were found to inhibit cell growth. Cultures were incubated overnight, X-gal (100 µg mL^−1^) was subsequently added, and cells were photographed after development of blue color indicating high β-galactosidase activity. Alternatively, ONPG was added to the cultures to quantify β-galactosidase activity by adapting the method previously described by Miller^54^. Three individual replicates of each assay condition were performed with an additional fourth replicate performed of the WT *nhaA*-I reporter for comparison to the three replicates of the M1 *nhaA*-I reporter.

### In vitro transcription assays

The transcription termination assays were conducted by adapting a previously established method for single-round transcription^55^. The DNA templates (Supplementary Table II in the Supplement) for the RNA transcripts (Fig. 5A) include the native promotor from *D. propionicus*. Transcription reactions were performed with 100 nM DNA template in 40 mM Tris–HCl (pH 7.5 at 23 °C), 100 mM KCl, 4 mM MgCl_2_, 0.01 mg mL^−1^ BSA, 1% (v/v) glycerol, and 0.04 U µL^−1^ *E. coli* RNA Polymerase, Holoenzyme (New England Biolabs). The *E. coli* RNA polymerase was supplied in a buffer containing NaCl, thus adding 4 mM NaCl to the final reaction. The transcription reaction was initiated in the presence of ApA dinucleotide (0.135 mM), GTP and ATP (2.5 µM each), UTP (1.0 µM), and [α-^32^P]-UTP (2 µCi). This initial reaction mixture was incubated for 10 min at 37 °C to allow RNA polymerase to stall at the first C residue of the RNA transcript, which occurs 16 nucleotides from the transcription start site. The halted complexes resumed transcription with the addition of an elongation mixture containing 150 µM each of GTP, ATP, and CTP and 30 µM UTP. Also, 0.1 mg mL^−1^ heparin was added at that time to prevent further RNA polymerase initiation, and the mixture was incubated for 30 min at 37 °C. It was necessary to add the additional monovalent ions (candidate riboswitch ligands) with the elongation mixture after the initiation complex was formed, otherwise there was a decrease in overall yield of transcription products.

All annotations for monovalent ion supplementation do not include 100 mM KCl present in the initial transcription reaction mixture in the buffer. For example, the reaction annotated as supplemented with 200 mM K^+^ has a total of 300 mM KCl. Transcription products were separated by denaturing 10% PAGE, imaged using a Typhoon FLA 9500 Molecular Scanner, and quantified using ImageQuant software.

Three replicate transcription termination experiments were performed on three separate days, and a representative of the resulting PAGE autoradiogram is presented (Fig. 5B, top). Using band intensities, the amounts of full-length (FL) and terminated (T) transcripts were estimated by accounting for the different number of U residues in each transcript, and the resulting values for all three experimental replicates are depicted in the plot (Fig. 5B, bottom). The number of U nucleotides in the initiation region for all constructs is 5. The number of U nucleotides in the elongation region is 45 for the WT and M2 constructs, 47 for the M3 construct, and 46 for the M4 construct. The percent of [α-^32^P]-UTP relative to total UTP concentration in the initiation and elongation reactions (7% and 0.2%, respectively) was established, and the relative amount of radioactivity per terminated (R_T_) and full length (R_FL_) transcripts was calculated for each transcript size using the following equation: [(Number of U residues in initiation region)(7%)] + [(Number of U residues in elongation region)(0.2%)] = R. R_T_/R_FL_ is equal to the correction factor (X%) that accounts for the increased number of radiolabeled U residues in the full-length transcript. The equation used to establish the percent of transcription termination was: 100{T/[T + (FL)(X%)]} = percent termination. The resulting values were used to establish the fraction of RNA transcripts that are full length, which was plotted (Fig. 5B). These methods were also used to establish the data for the M4 construct (Fig. 5C).

## Supplementary Information

Supplementary Information 1.

Supplementary Information 2.

## Data Availability

The datasets generated and/or analysed during the current study are available in the Rfam repository, [https://rfam.xfam.org/family/RF03057#tabview=tab1 and https://rfam.xfam.org/family/RF03038#tabview=tab1]. All other data needed to evaluate the author’s conclusions are presented in the main or Supplementary Materials sections.
