# Supplementary material for: Lithium-sensing riboswitch classes regulate expression of bacterial cation transporter genes
Source: Sci Rep. 2022 Nov 9;12:19145. doi: 10.1038/s41598-022-20695-6 (PMC9646797; doi:10.1038/s41598-022-20695-6)
Supplement: Supplementary file 1 — Supplementary Information 1. [file 41598_2022_20695_MOESM1_ESM.docx]

Supplementary Material

**Lithium-sensing riboswitch classes regulate expression of bacterial cation transporter genes**

**Neil White^1,2^, Harini Sadeeshkumar^1^, Anna Sun^1^, Narasimhan Sudarsan^2^, Ronald R. Breaker^1,2,3*^**

^1^Department of Molecular, Cellular and Developmental Biology, Yale University, New Haven, Connecticut 06520-8103, USA

^2^Howard Hughes Medical Institute, Yale University, New Haven, CT 06520-8103, USA

^3^Department of Molecular Biophysics and Biochemistry, Yale University, New Haven, Connecticut 06520-8103, USA

*Corresponding author. Email: [ronald.breaker@yale.edu](mailto:ronald.breaker@yale.edu)


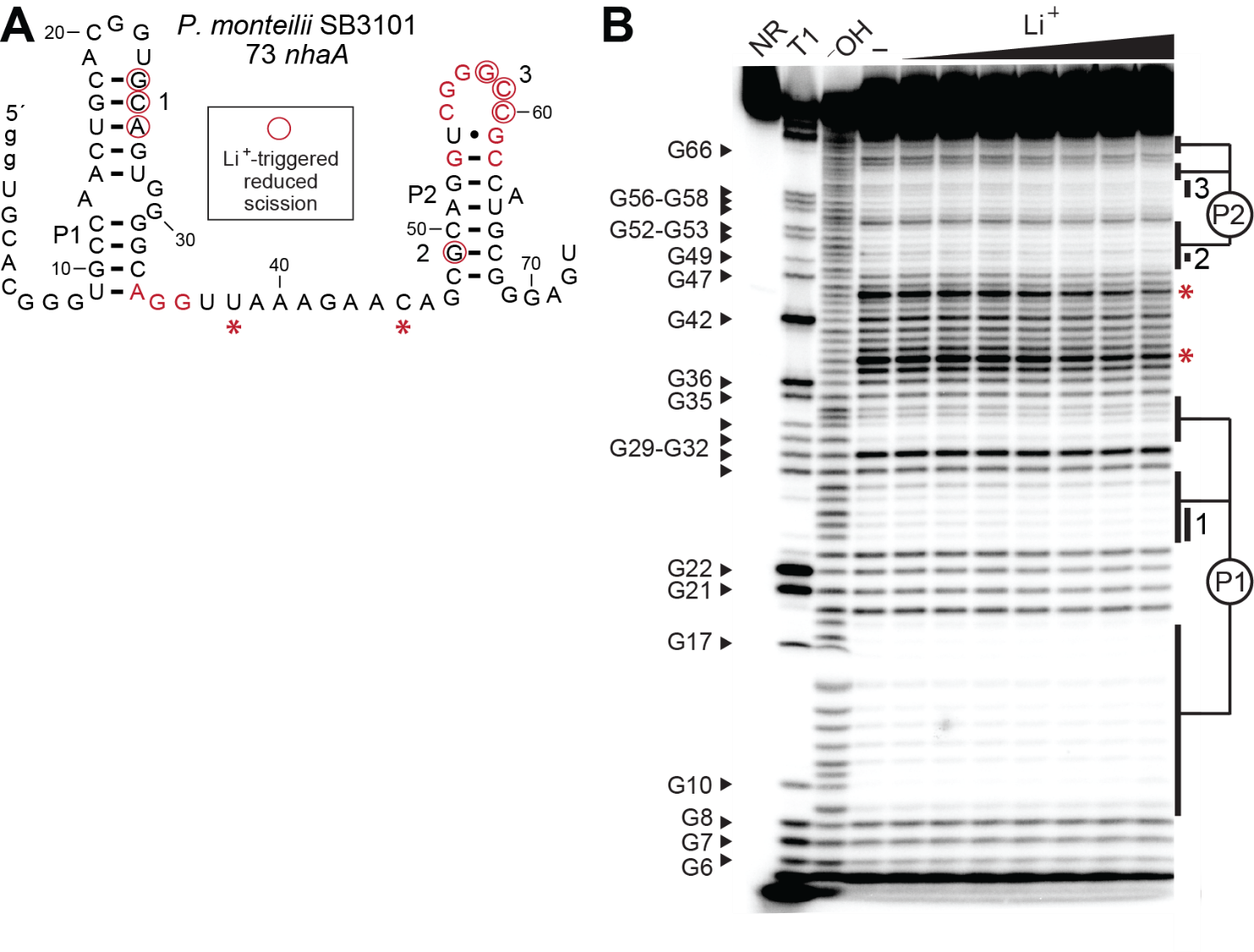


**Supplementary Figure I.** **Li^+^-induced structural modulation the 73 *nhaA* RNA construct evaluated with an in-line probing gel depicted in low intensity.** (**A**) Sequence and secondary structure model for the Li^+^-I riboswitch aptamer construct 73 *nhaA* from the bacterium *Pseudomonas monteilii* designating two sites (nucleotides 38 and 45, see asterisks) that undergo the greatest intensity changes upon addition of Li^+^. Other annotations are as described for **Fig. 4A**. (**B**) Autoradiogram of the separation of products from in-line probing reactions by PAGE. The image and annotations are as depicted in **Fig. 4B** except that the image is depicted at lower intensity and the sites of greatest band modulation are designated with asterisks. These differences are visually obscured on the high-intensity image in **Fig. 4B** that is further discussed in the main text.


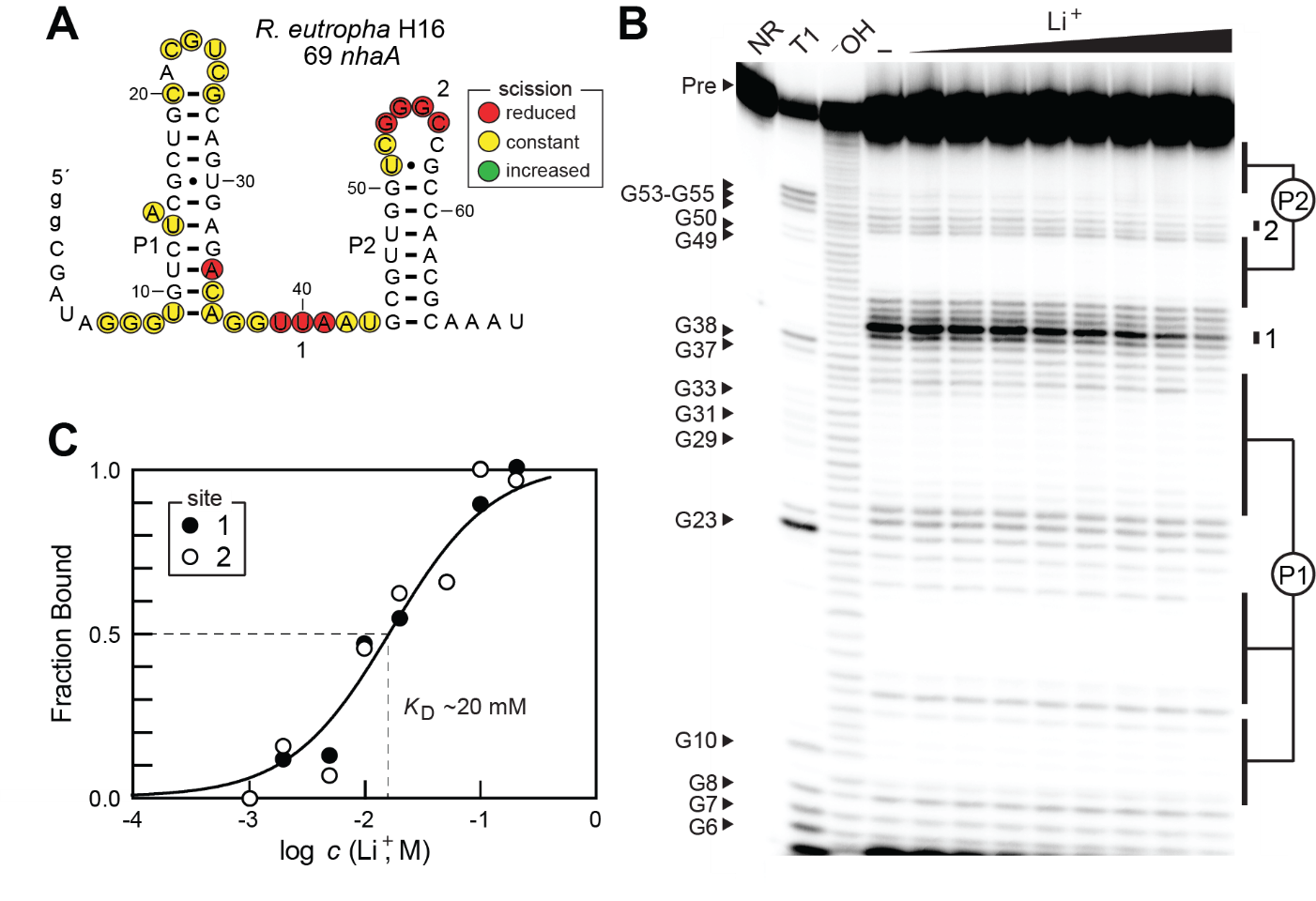


**Supplementary Figure II.** **Li^+^ induces structural modulation of a Li^+^-I aptamer from *R. eutropha* H16.** (**A**) Sequence and secondary structure model for the Li^+^-I riboswitch aptamer construct 69 *nhaA* derived from the *nhaA* gene from *Ralstonia eutropha*. Additional annotations are as described for **Fig. 4**. (**B**) PAGE analysis of in-line probing reactions with 5′ ^32^P-labeled 69 *nhaA* RNA in the absence of Li^+^ (–), or in the presence of Li^+^ concentrations ranging from 2 mM to 200 mM. NR, T1 and ^–^OH identify RNAs subjected to no reaction, partial digestion with RNase T1 (cleaves after G nucleotides) and partial digestion with hydroxide (cleaves after each nucleotide). (**C**) Plot of the estimated fraction of RNAs bound to Li^+^ versus the logarithm of the Li^+^ concentration. The solid line depicts a theoretical 1-to-1 binding curve with a *K*_D_ of no lower than 20 mM.

**
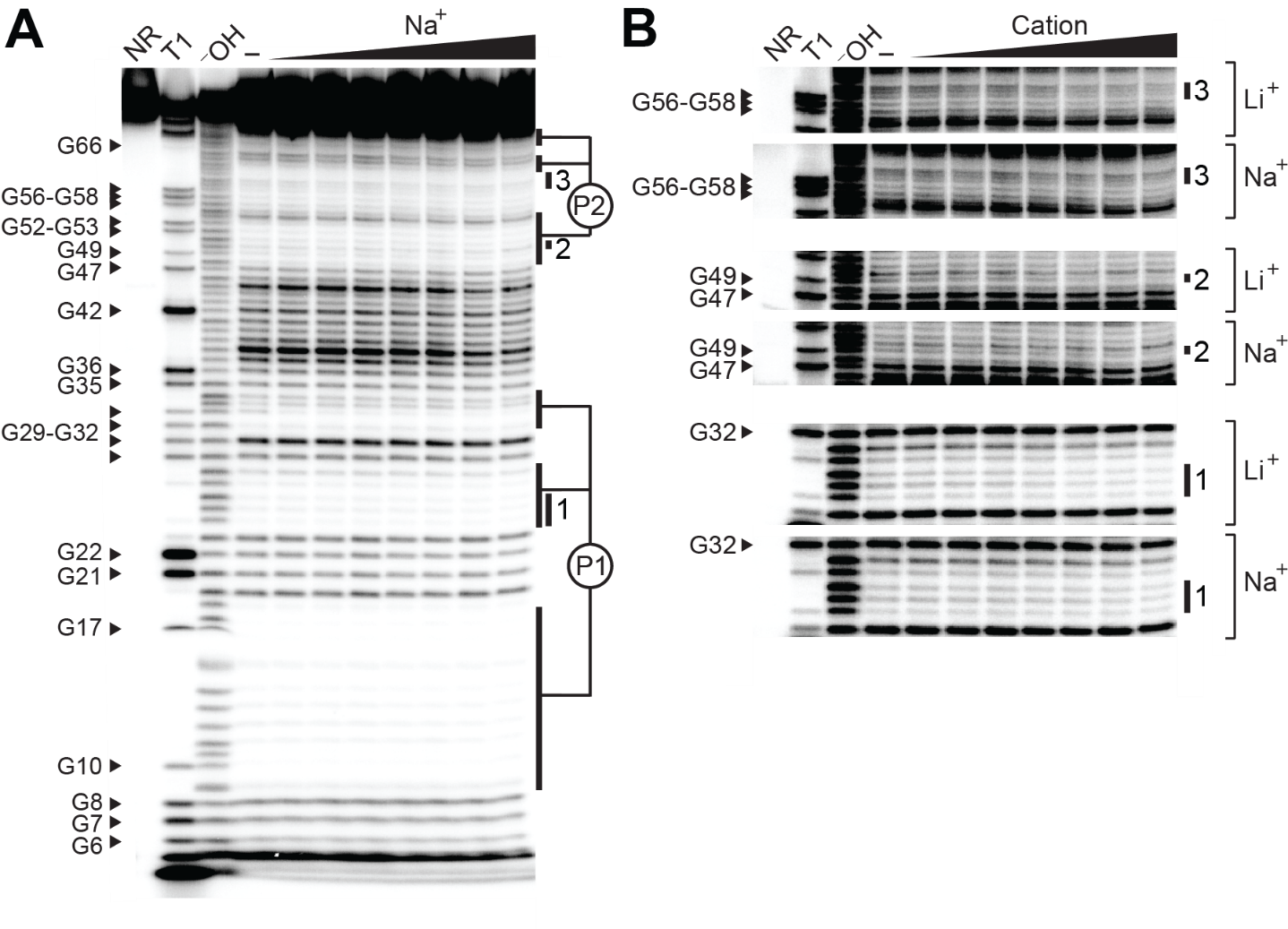
**

**Supplementary Figure III. The 73 *nhaA* RNA construct representing a Li^+^-I riboswitch aptamer selectively responds to Li^+^ compared to Na^+^.** (**A**) PAGE analysis of in-line probing reactions with 5′ ^32^P-labeled 73 *nhaA* RNA (**Fig. 4A**) in the presence of Na^+^ ranging from 2 mM to 200 mM. Other annotations are as described for **Fig. 4B**. (**B**) Comparison of modulation sites 1, 2 and 3 using image excerpts of the corresponding regions of the PAGE images presented in **Fig. 4B** and in A of this figure. The dynamic ranges for pixel intensities have been adjusted to highlight the band intensity differences between Li^+^ and Na^+^ in-line probing assays.


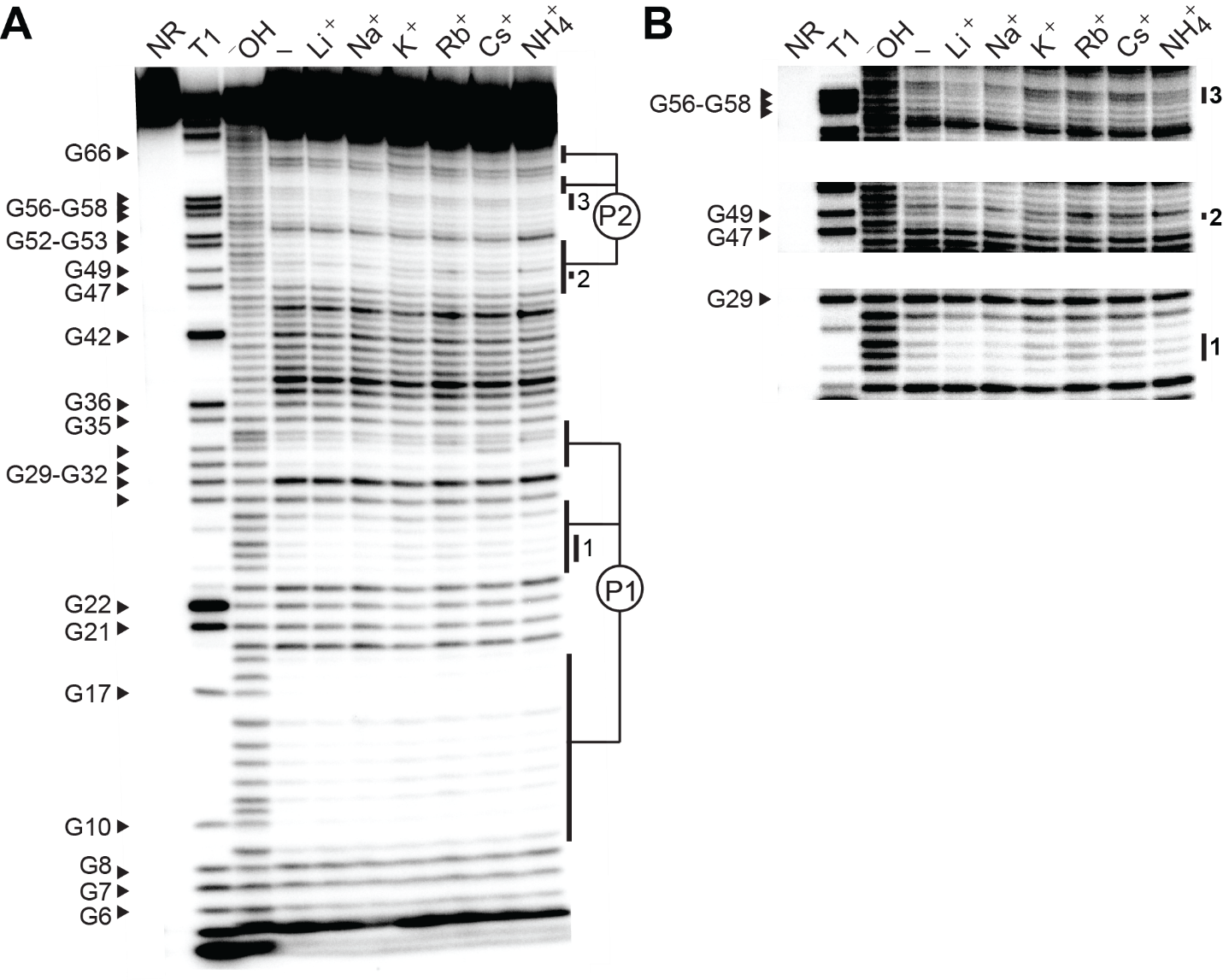


**Supplementary Figure IV. The 73 *nhaA* RNA construct representing a Li^+^-I riboswitch aptamer favors Li^+^ binding compared to other alkali metal cations and ammonium.** (**A**) PAGE analysis of in-line probing reactions with 5′ ^32^P-labeled 73 *nhaA* RNA (**Fig. 4A**) in the presence of 200 mM of the cations designated, except Rb^+^ was tested at 50 mM due to its lower solubility. Other annotations are as described for **Fig. 4B** and **Fig. 4D**. (**B**) Comparison of modulation sites 1, 2 and 3 using image excerpts of the corresponding regions of the PAGE images presented in A. Note that Li^+^ reduces band intensity the most, whereas Na^+^ and NH_4_^+^ exhibit less suppression of band intensity at these sites. This indicates that Na^+^ and NH_4_^+^ likely bind with poorer affinities, which is consistent with their failure to activate reporter gene expression (**Fig. 2**).


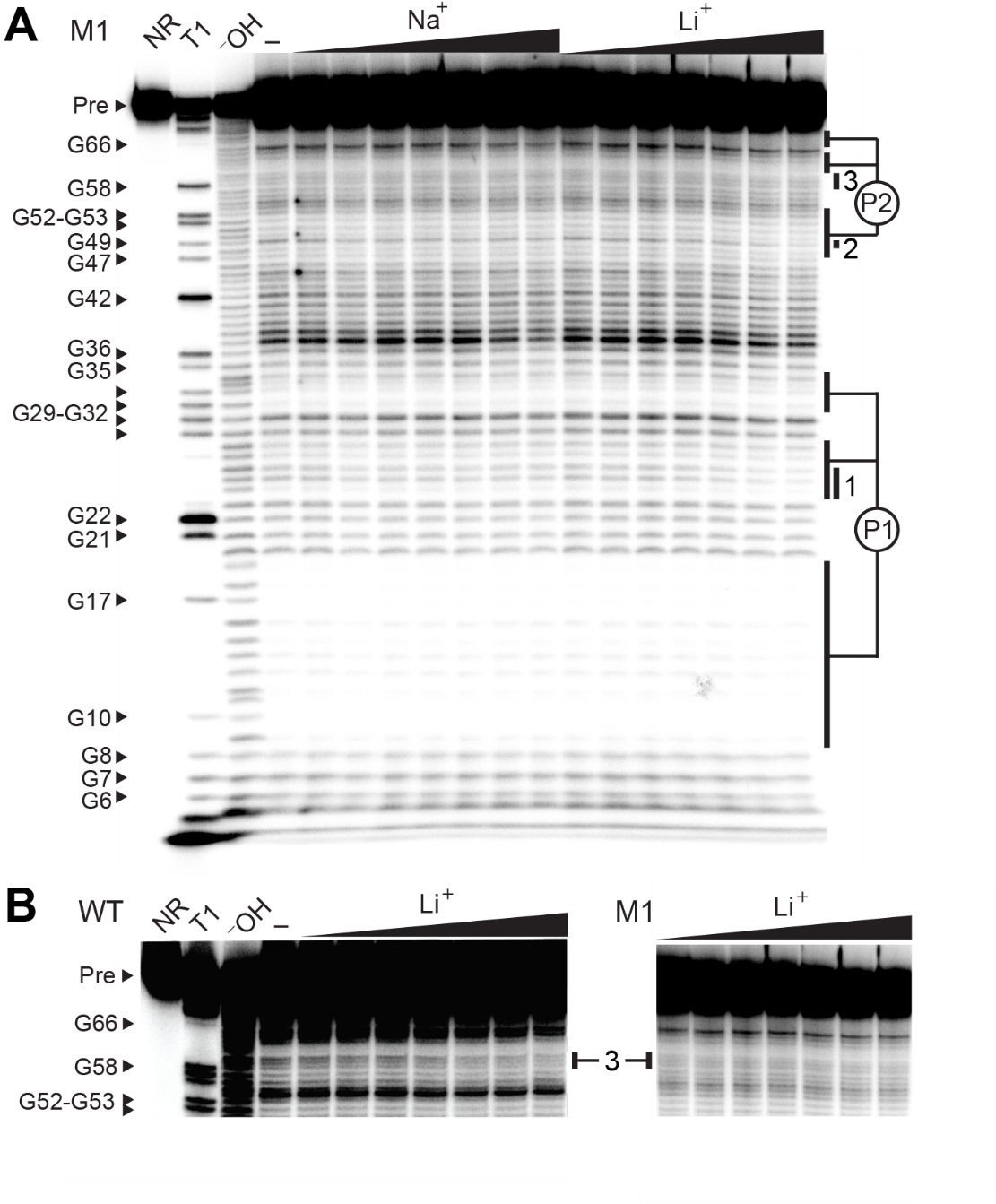


**Supplementary Figure V. Mutation of strictly conserved nucleotides disrupts Li^+^ binding to a Li^+^-I riboswitch aptamer.** (**A**) PAGE analysis of in-line probing reactions of the M1 version of the 73 *nhaA* construct (**Fig. 4A**) in the absence of ligand (–), or in the presence of Na^+^ or Li^+^ concentrations ranging from 2 mM to 200 mM. Other annotations are as described for **Fig. 4B**. Notes: (i) The bands in the T1 lane corresponding to nucleotide positions 65 and 57 are absent due to their mutation to A nucleotides; (ii) although incomplete modulation of sites 1 through 3 occur with Li^+^, this response occurs at a higher Li^+^ concentration than is observed for the WT 73 *nhaA* construct, suggesting the mutations have weakened affinity for the ligand. (B) Side-by-side comparison of the in-line probing results near site 3 (labeled) for the WT (left) and M1 (right) constructs, demonstrating loss of Li^+^ responsiveness of bands at site 3 of M1. Data is from **Fig. 4B** and part A of this figure.


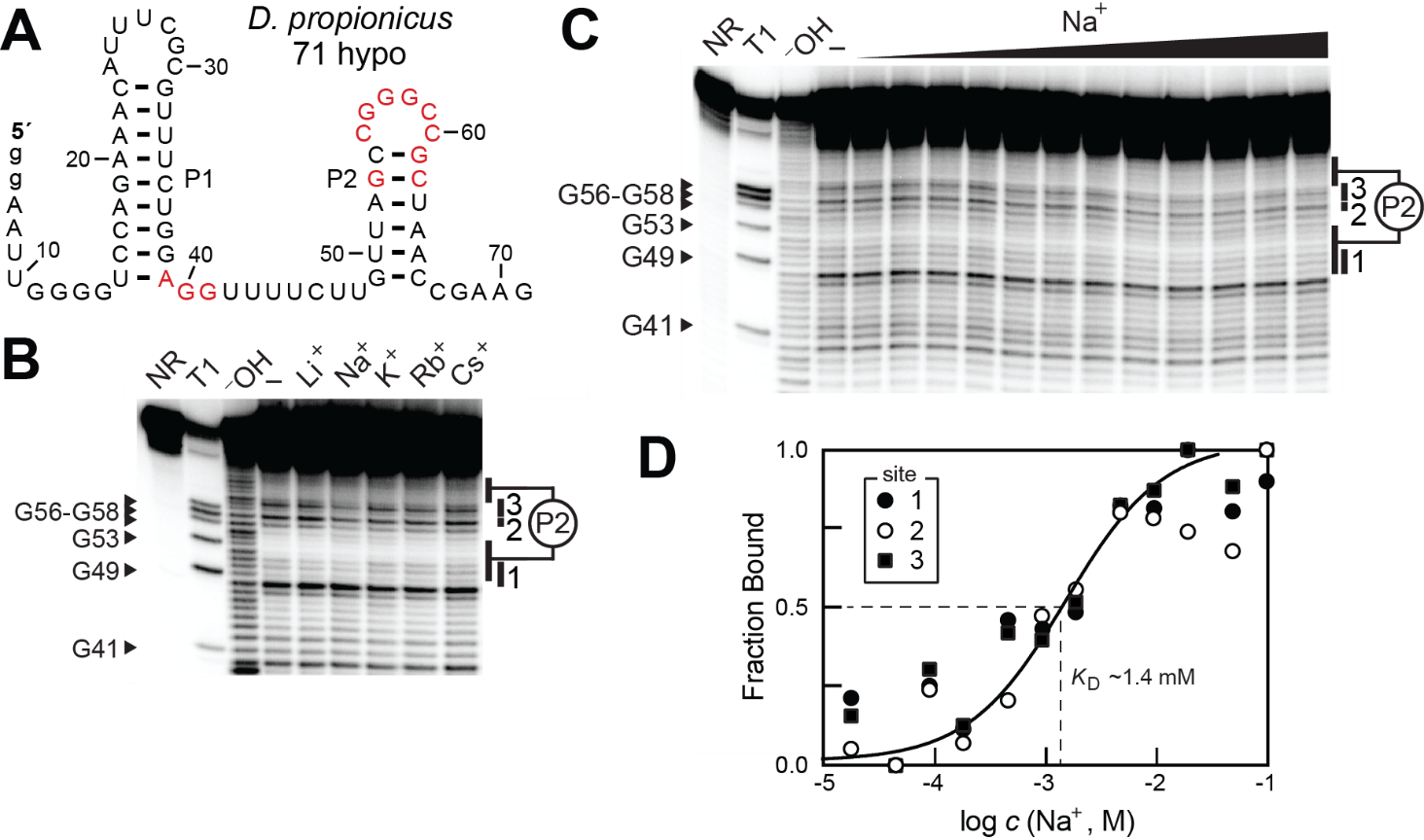


**Supplementary Figure VI. In-line probing analyses of a Na^+^-responsive variant of *nhaA*-I motif RNAs.** (**A**) Sequence and secondary structure model for the 71 hypo RNA construct from *D. propionicus*. Annotations are as described for **Fig. 2A**. (**B**) PAGE analysis of in-line probing reactions of the 5́ ^32^P-labeled 71 hypo RNA construct in the absence (–) or presence of 100 mM alkali metal cations (Rb^+^ was tested at 50 mM). Other annotations are as described for **Fig. 4B**. (**C**) In-line probing assay results for the 71 hypo RNA construct incubated with various concentrations of Na^+^ ranging from 20 μM to 100 mM. (**D**) Plot of the estimated fraction of RNAs bound to Na^+^ versus the logarithm of the concentration of Na^+^. The solid line depicts a theoretical 1-to-1 binding curve with a *K*_D_ of ~1.4 mM. Note that data points most likely vary from the theoretical curve due to the modest modulation of band intensities, which is typical of Na^+^ riboswitches reported previously (*18*).


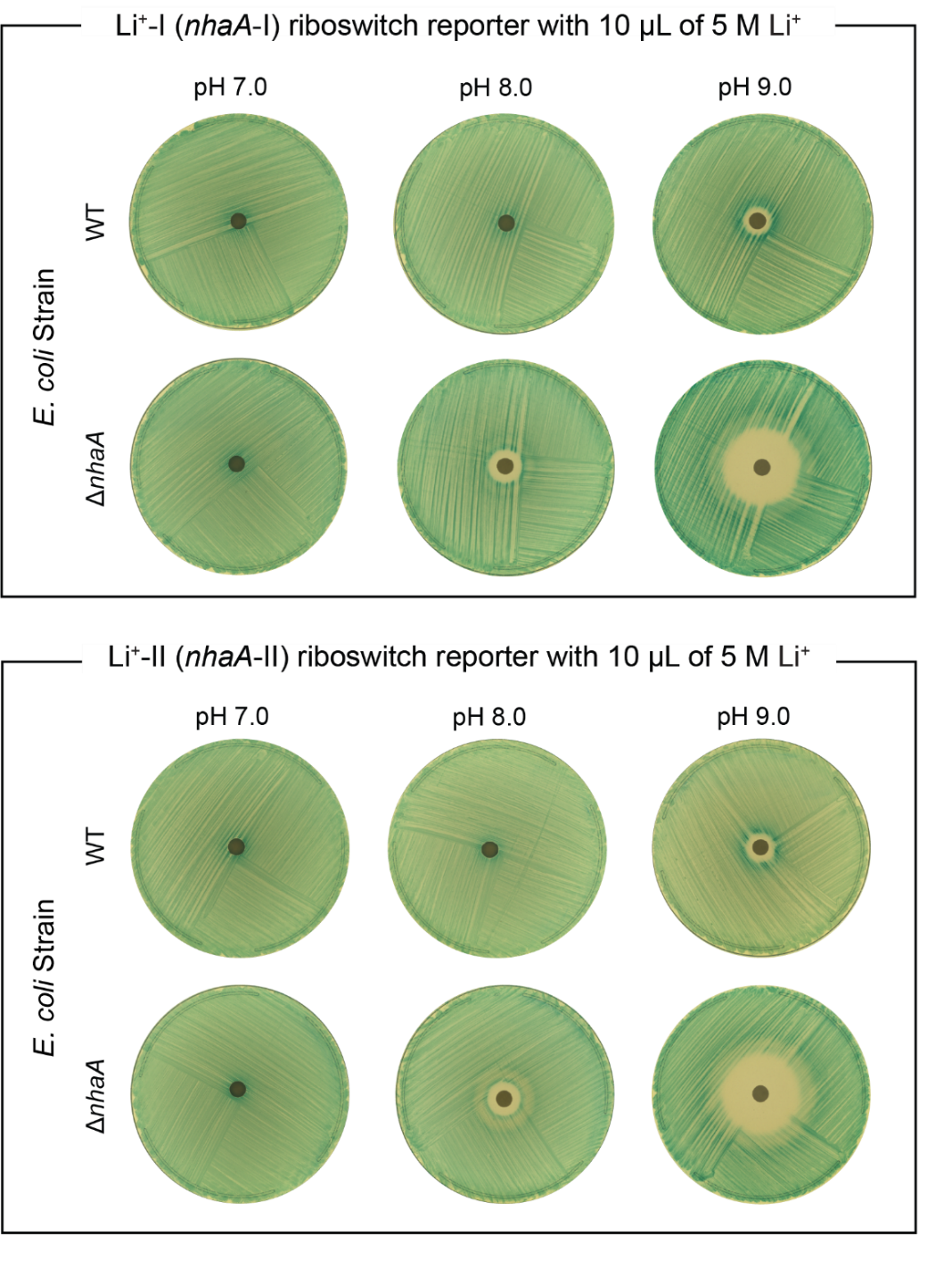


**Supplementary Figure VII. Lithium causes increased toxicity when cells are grown with alkaline pH media.** Top: WT and Δ*nhaA* *E. coli* strains carrying a Li^+^-I (*nhaA*-I) riboswitch-reporter fusion construct (**Fig. 2A**) were grown on LBK agar plates containing X-gal (100 µg mL^-1^) and carbenicillin (100 µg mL^-1^) and were buffered at pH 7.0 (100 mM PIPES), pH 8.0 (100 mM TAPS), or pH 9.0 (100 mM AMPSO) as indicated. All filter disks received 10 µL of 5 M LiCl prior to placement and the plates were incubated overnight before photographing. Bottom: WT and Δ*nhaA* *E. coli* strains carrying a Li^+^-II (*nhaA*-II) riboswitch-reporter fusion construct (**Fig. 2D**) were analyzed as described above. See the main text and Methods section for additional details. Note that the pH 9.0 plates are the images depicted in **Fig. 2B** and **Fig. 2E**.


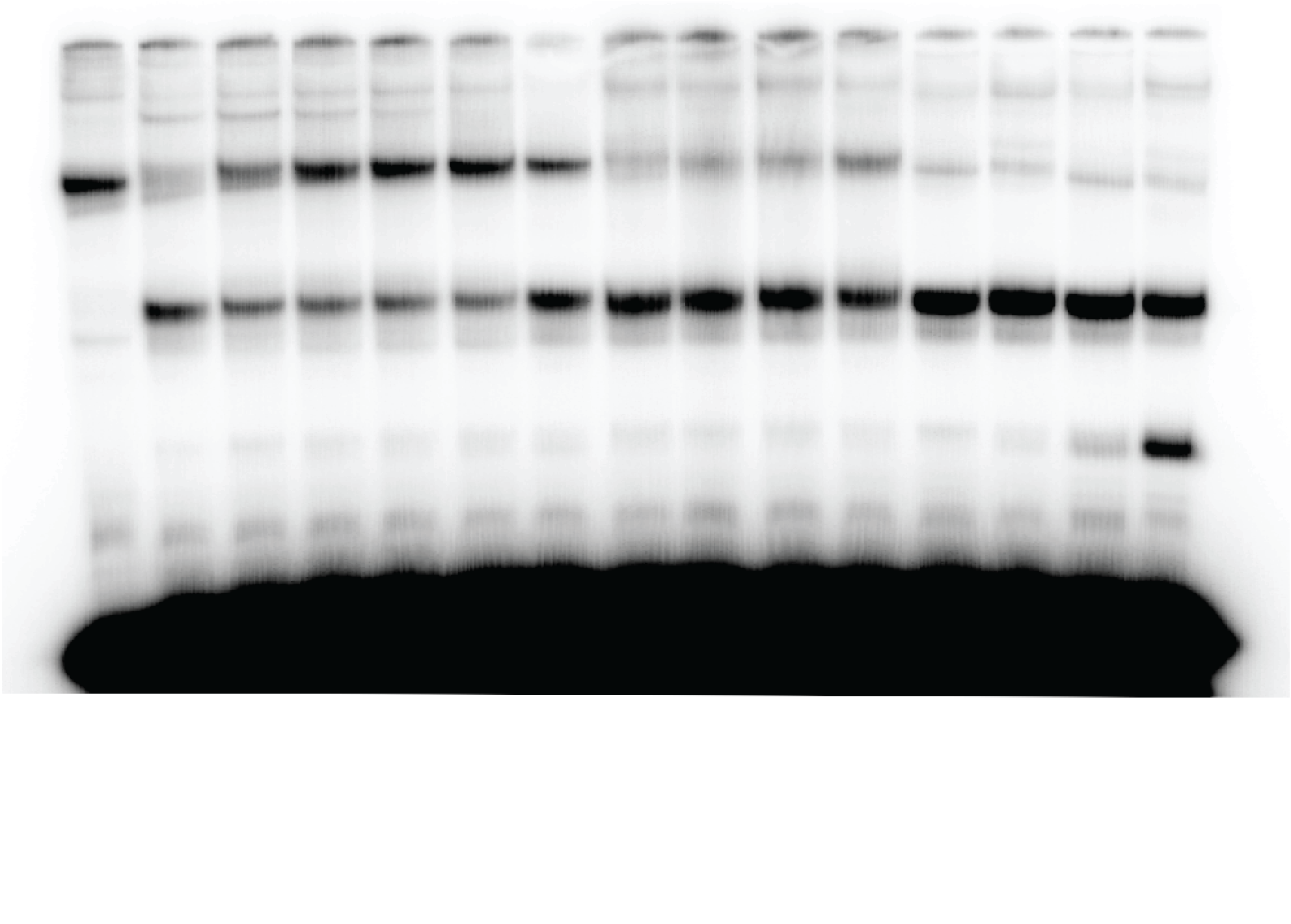


**Supplementary Figure VIII. Uncropped version of the gel image in Fig. 5B.** Key bands are identified and labeled in Fig. 5B. The top-most bands correspond to the wells of the gel.


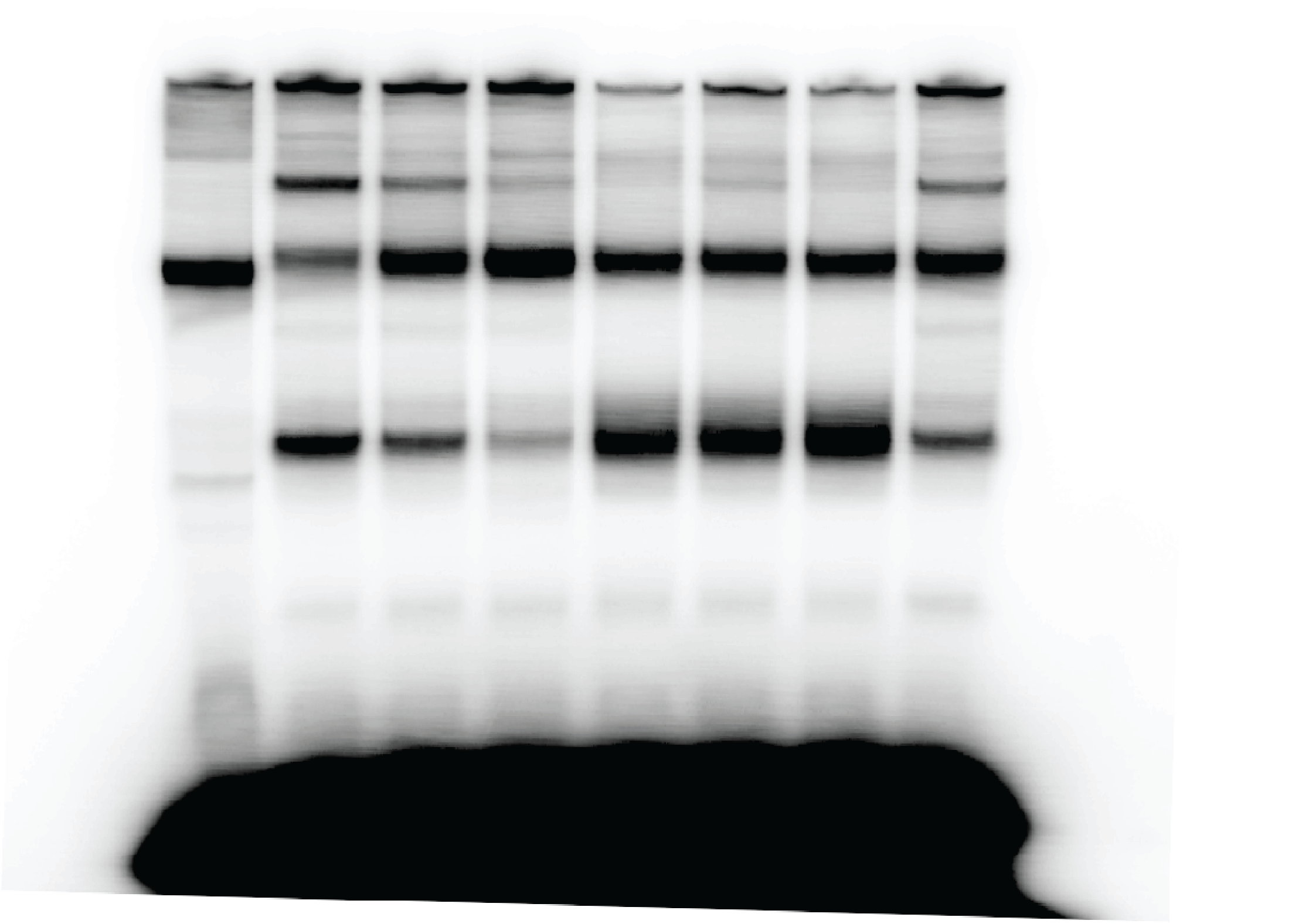


**Supplementary Figure IX. Uncropped version of the gel image in Fig. 5C.** Key bands are identified and labeled in Fig. 5C. The top-most bands correspond to the wells of the gel.

**Supplementary Table I.** Gene associations for ORFs located downstream of *nhaA*-I and *nhaA*-II motif RNAs. Gene names and presumed protein function as annotated for *nhaA*-I (left) and *nhaA*-II (right) motif RNAs are listed along with the number uncovered (Count) in the genomic DNA sequence databases searched. When available, gene names are listed in bold, italic font. “No gene information” indicates that the database lacks information on the downstream gene, as frequently occurs in metagenomic DNA sequence datasets. “None” indicates no gene name was annotated. “Hypothetical” annotation indicates an ORF is predicted but that no protein homolog with an established function is available for comparison. This category could include several different protein types. “Other” includes all gene annotations that occur fewer than three times. “Putative” annotation designates the presence of an ORF whose protein product is predicted to exhibit the function provided, but whose exact function has not been experimentally determined. “DUF” indicates a protein domain of unknown function. DUF1646 has been proposed to function as a Na^+^/H^+^ antiporter: [CDD Conserved Protein Domain Family: DUF1646 (nih.gov)](https://www.ncbi.nlm.nih.gov/Structure/cdd/cddsrv.cgi?ascbin=8&maxaln=10&seltype=2&uid=pfam07854).

| ***nhaA*-I Motif RNAs** | | |  | ***nhaA*-II Motif RNAs** | | |
| --- | --- | --- | --- | --- | --- | --- |
|  |  |  |  |  |  |  |
| **Gene Annotation** | **Protein Function Annotation** | **Count** |  | **Gene Annotation** | **Protein Function Annotation** | **Count** |
| ***nhaA*** | Na^+^(Li^+^)/H^+^ antiporter | 356 |  | ***nhaA*** | Na^+^(Li^+^)/H^+^ antiporter | 36 |
| No gene information | Not applicable | 185 |  | No gene information | Not applicable | 2 |
| [Hypothetical] | Various unknown | 171 |  |  |  |  |
| None | Sodium/proton antiporter | 130 |  |  |  |  |
| Other | Various | 52 |  |  |  |  |
| ***otsA*** | Trehalose-6-phosphate synthase/phosphatase | 16 |  |  |  |  |
| ***nhaD*** | Na^+^/H^+^ antiporter | 6 |  |  |  |  |
| [Putative] | Citrate transporter | 5 |  |  |  |  |
| [Putative] | Glycosyl transferase | 5 |  |  |  |  |
| None | Cation/H^+^ antiporter | 4 |  |  |  |  |
| [DUF1646] | Proposed Na^+^/H^+^ antiporter | 4 |  |  |  |  |
| ***trkA*** (or *TrkA*-N) | K^+^ uptake (binds NAD^+^ and NADH) | 4 |  |  |  |  |
| ***mscS*** | Mechanosensitive ion channel protein | 3 |  |  |  |  |
| ***oadG*** | Oxaloacetate decarboxylase | 3 |  |  |  |  |
|  | Total | 944 |  |  | Total | 38 |

**Supplementary Table II.** Synthetic DNAs. Underlined, italicized nucleotides designate mutations relative to the WT sequence. Red nucleotides represent the T7 RNA polymerase promoter sequence.

**DNA Templates for Production of RNA Constructs for In-line Probing Assays**

*P. monteilii SB3101* 73 *nhaA* (WT):

TAATACGACTCACTATAGGTGCACGGGTGCCAACTGCACGGTGCAGTGGGGCAGGTTAAAGAACAGCGCAGGTCGGGCCGCCATGCGGGAGT

Forward primer:

TAATACGACTCACTATAGGTGCACGGGTGCCAACTGCACGGTGCAGTGGGGCAGGTTAAAGAACAG

Reverse primer:

ACTCCCGCATGGCGGCCCGACCTGCGCTGTTCTTTAACCTGCCCCACTGCACCGTGCAGTTGGCAC

(M1):

Forward primer:

TAATACGACTCACTATAGGTGCACGGGTGCCAACTGCACGGTGCAGTGGGGCCAGTTAAAGAACAG

Reverse primer: ACTCCCGCATGGCGGCCCGACCTGCGCTGTTCTTTAACTGGCCCCACTGCACCGTGCAGTTGGCAC

*R. eutropha* H16 69 *nhaA*

TAATACGACTCACTATAGGCGATAGGGTGTCTACGCTGCACGTCGCAGTGAGACAGGTTAATGCGTTGGTCGGGCCGCCAACGCAAAT

Forward primer:

TAATACGACTCACTATAGGCGATAGGGTGTCTACGCTGCACGTCGCAGTGAGACAGGTTAATGC

Reverse primer:

ATTTGCGTTGGCGGCCCGACCAACGCATTAACCTGTCTCACTGCGACGTGCAGCGTAGACACCC

*D. propionicus* 71 hypo

TAATACGACTCACTATAGGAATTGGGGTCCAGAAACATTTCGCGTTTCTGGAGGTTTTCTTGTTAGCCGGGCCGCTAACCGAAG

Forward primer:

TAATACGACTCACTATAGGAATTGGGGTCCAGAAACATTTCGCGTTTCTGGAGGTTTTCTTGTT

Reverse primer:

CTTCGGTTAGCGGCCCGGCTAACAAGAAAACCTCCAGAAACGCGAAATGTTTCTGGACCCCAAT

**DNA Oligonucleotides for Use as Genetic Constructs**

*nhaA*-I Riboswitch

GCTCGAGCTCCGTACGACGAATTCCAAAAATAATGTTGATCCTTTTAAATAAGTCTGATAAAATGTGAACTAAGGAAGATCATAAGGGCATGGGTGCGCGATCCCCTATTGGATCGGCAGGTTGTTGCGCTGGTCGGGCCGCCAGCGTTGGAGACCTGCATGTATCATCGCGCGGATACGAGCGCCGTCGCTCGTCCTTTGCTTCATAAGCGGATTTCTTCCTCCCATGCGGGAGGACGCCCATGAGCGGGCCGTCCCGGACCCAGGATCCAAAGGA

*nhaA*-II Riboswitch

TACGACGAATTCCAAAAATAATGTTGATCCTTTTAAATAAGTCTGATAAAATGTGAACTAAGGCCCCAGCGATCAGGTCCGGGCATGCCCCGGGACCAGGCCGGCGCCCCAGGGCGAACCCGGTCAGATGCCCGTCGATAACCGGGCGTCACGGAATACAGACCCCCGGACCGGAGGCCGCCGTGGCGCGCAGACTGACACTCGACTTCCTGGATCCAAAGGA

Genetic Constructs Forward Primer

TACGACGAATTCCAAAAATAATGTTGATCC

*nhaA*-I Riboswitch Reverse Primer

TCCTTTGGATCCTGGGTCCG

*nhaA*-II Riboswitch Reverse Primer

TCCTTTGGATCCAGGAAGTC

**DNA Oligonucleotides for Use as Transcription Templates**

*D. propionicus* Hypo Riboswitch Transcription Template (WT)

TACGACGAATTCCTCAATTTCTATTGATAAAAGAGCTTCTGTCCATTATAAAAAGAAAAATAAATAATAATTGGGGTCCAGAAACATTTCGCGTTTCTGGAGGTTTTCTTGTTAGCCGGGCCGCTAACCGAAGCGTAAATGCTTCTGTTAGCGGCCTTTTTTTTATCTTTATGCGGGAAAAAACATAATTAATAGCTCCCTAACTGAAGCGAATGGATCCAAAGGA

*D. propionicus* Hypo Riboswitch Transcription Template (M2)

TACGACGAATTCCTCAATTTCTATTGATAAAAGAGCTTCTGTCCATTATAAAAAGAAAAATAAATAATAATTGGGGTCCAGAAACATTTCGCGTTTCTGGA*AA*TTTTCTTGTTAGCCGGGCCGCTAACCGAAGCGTAAATGCTTCTGTTAGCGGCCTTTTTTTTATCTTTATGCGGGAAAAAACATAATTAATAGCTCCCTAACTGAAGCGAATGGATCCAAAGGA

*D. propionicus* Hypo Riboswitch Transcription Template (M3)

TACGACGAATTCCTCAATTTCTATTGATAAAAGAGCTTCTGTCCATTATAAAAAGAAAAATAAATAATAATTGGGGTCCAGAAACATTTCGCGTTTCTGGAGGTTTTCTTGTTAGC*TT*GGCCGCTAACCGAAGCGTAAATGCTTCTGTTAGCGGCCTTTTTTTTATCTTTATGCGGGAAAAAACATAATTAATAGCTCCCTAACTGAAGCGAATGGATCCAAAGGA

*D. propionicus* Hypo Riboswitch Transcription Template (M4)

TACGACGAATTCCTCAATTTCTATTGATAAAAGAGCTTCTGTCCATTATAAAAAGAAAAATAAATAATAATTGGGGTCCAGAAACATTTCGCGTTTCTGGAGGTTTTCTTGTTAG*T*CGGGCCGCTAACCGAAGCGTAAATGCTTCTGTTAGCGGCCTTTTTTTTATCTTTATGCGGGAAAAAACATAATTAATAGCTCCCTAACTGAAGCGAATGGATCCAAAGGA

*D. propionicus* Hypo Riboswitch Transcription Template (FL Marker)

TACGACGAATTCCTCAATTTCTATTGATAAAAGAGCTTCTGTCCATTATAAAAAGAAAAATAAATAATAATTGGGGTCCAGAAACATTTCGCGTTTCTGGAGGTTTTCTTGTTAGCCGGGCCGCTAACCGAAGCGTAAATGCTTCTGTTAGCGGCC*AAAAAAAA*ATCTTTATGCGGGAAAAAACATAATTAATAGCTCCCTAACTGAAGCGAATGGATCCAAAGGA

*D. propionicus* Hypo Riboswitch Transcription Template Forward Primer

TACGACGAATTCCTCAATTTCTATTGATAA

*D. propionicus* Hypo Riboswitch Transcription Template Reverse Primer

TCCTTTGGATCCATTCGCTTCAGTTAGGG
